# The molecular basis of thioalcohol production in human body odour

**DOI:** 10.1038/s41598-020-68860-z

**Published:** 2020-07-27

**Authors:** Michelle Rudden, Reyme Herman, Matthew Rose, Daniel Bawdon, Diana S. Cox, Eleanor Dodson, Matthew T. G. Holden, Anthony J. Wilkinson, A. Gordon James, Gavin H. Thomas

**Affiliations:** 1 Department of Biology, University of York, Wentworth Way, York, YO10 5DD UK; 2 Unilever R&D, Colworth Science Park, Sharnbrook, Bedford, MK44 1LQ UK; 3 Department of Chemistry, University of York, Wentworth Way, York, YO10 5DD UK; 4 School of Medicine, University of St Andrews, St Andrews, KY11 9TF UK

**Keywords:** X-ray crystallography, Microbiology

## Abstract

Body odour is a characteristic trait of *Homo sapiens*, however its role in human behaviour and evolution is poorly understood. Remarkably, body odour is linked to the presence of a few species of commensal microbes. Herein we discover a bacterial enzyme, limited to odour-forming staphylococci that are able to cleave odourless precursors of thioalcohols, the most pungent components of body odour. We demonstrated using phylogenetics, biochemistry and structural biology that this cysteine-thiol lyase (C-T lyase) is a PLP-dependent enzyme that moved horizontally into a unique monophyletic group of odour-forming staphylococci about 60 million years ago, and has subsequently tailored its enzymatic function to human-derived thioalcohol precursors. Significantly, transfer of this enzyme alone to non-odour producing staphylococci confers odour production, demonstrating that this C-T lyase is both necessary and sufficient for thioalcohol formation. The structure of the C-T lyase compared to that of other related enzymes reveals how the adaptation to thioalcohol precursors has evolved through changes in the binding site to create a constrained hydrophobic pocket that is selective for branched aliphatic thioalcohol ligands. The ancestral acquisition of this enzyme, and the subsequent evolution of the specificity for thioalcohol precursors implies that body odour production in humans is an ancient process.

## Introduction

**Figure 1:**
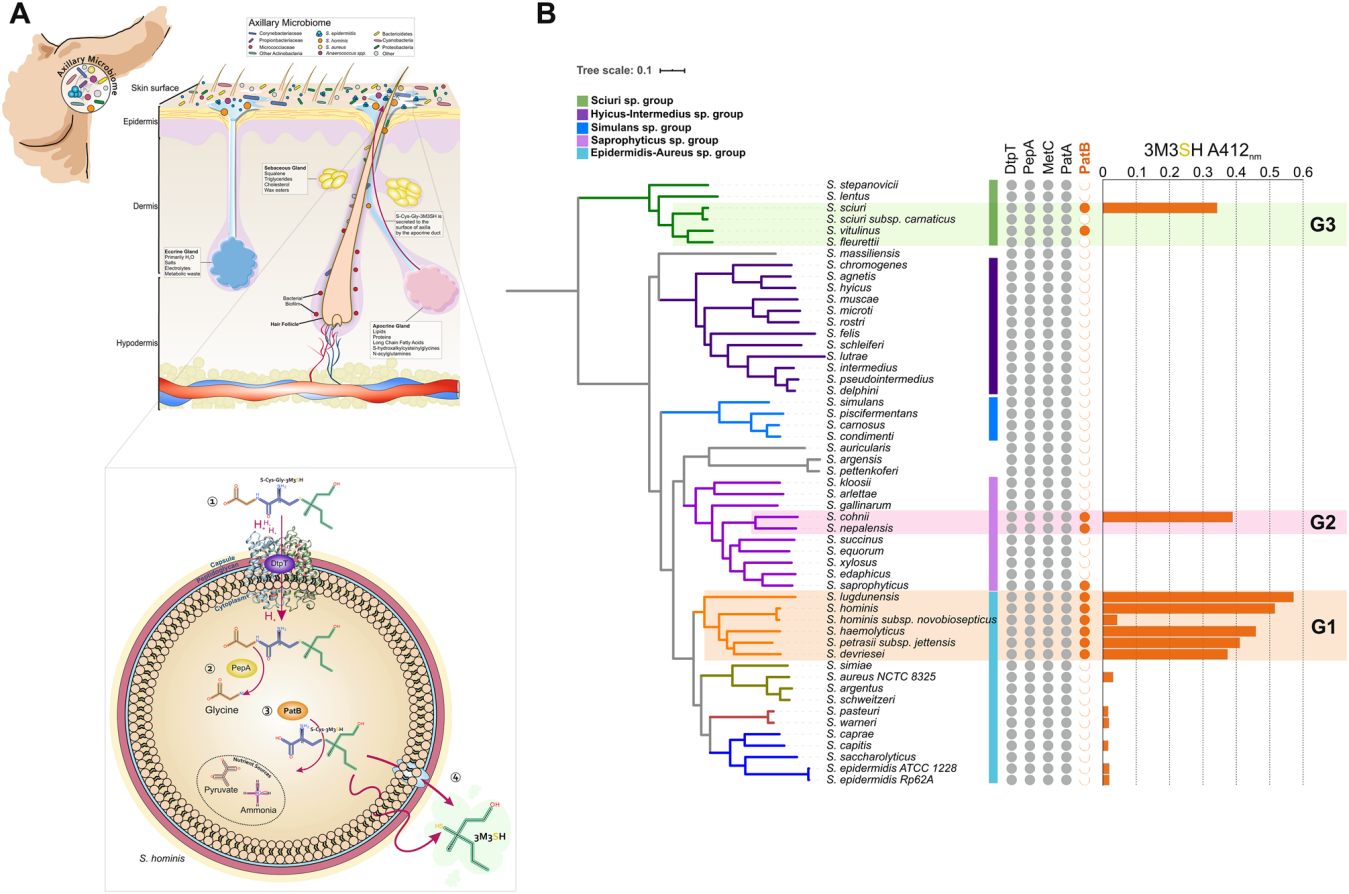
A unique clade of *Staphylococcus* spp. produce human malodour. (**A**) Overview of bacterial biotransformation of the odourless physiological malodour precursor Cys-Gly-3M3SH. Cys-Gly-3M3SH is secreted onto the surface of skin by axillary apocrine glands, subcutaneously located in the hypodermis. In *Staphylococcus hominis*, Cys-Gly-3M3SH is actively transported by the di-/tri-peptide transporter (DtpT) along with the movement of protons (1). Once inside the cell the terminal glycine is cleaved by a dipeptidase (PepA) to release Cys-3M3SH (2), which is metabolised by a C-S β-lyase liberating volatile 3M3SH (3), which diffuses or is exported out of the cell (4). The catabolism of Cys-Gly-3M3SH provides carbon and nitrogen as nutritional incentive in the form of glycine, ammonia and pyruvate. (**B**) Core genome phylogeny of staphylococcal species. Maximum Likelihood (ML) phylogenetic tree based on 53 representative species of staphylococci. Coloured strips represent the species group indicated in the colour key. Presence or absence of PLP-DE genes is indicated by filled (present)/unfilled (absent) circles. DtpT: di-/tri- peptide transporter; PepA: Aminopeptidase A; MetC: Cystathionine-β-lyase; PatA: Aspartate Aminotransferase; PatB: Putative Cysteine-S-conjugate β-lyase. Bars represent in vivo biotransformation of Cys-Gly-3M3SH by *Staphylococcus* spp. Biotransformation is quantified by release of 3M3SH labelled with DTNB and measured by absorbance at 412 nm. Groups of staphylococcal PatB enzymes are denoted by G1, G2 and G3. Phylogenetic tree and bar chart was generated using iTOL (https://itol.embl.de/).

Human body odour is produced by bacterial transformation of odourless precursor molecules secreted onto the surface of the skin by apocrine glands^1-3^. These glands are one of two major types of sweat gland found in *Homo sapiens*, the other being the eccrine glands. Eccrine glands are found in high density all over the body, they open directly onto the surface of the skin and are essential for thermoregulation^4^ (Fig. 1A). In contrast, apocrine glands open into hair follicles and typically occur in high density at specific body sites (axilla [underarm], nipple and external genitalia) (Fig. 1A); their exact function and physiological role in modern humans remain poorly understood. The axillary microbiota plays an important role in the generation of human body odour. *Staphylococcus*, *Cutibacterium* (formerly *Propionibacterium*) and *Corynebacterium* are the dominant genera colonizing the axilla^5,6^, with recent metataxonomic studies highlighting the additional presence of Gram-positive anaerobic cocci (GPAC), notably *Anaerococcus* and *Peptoniphilus* species^5,7^. Human axillary malodour is comprised of a mixture of volatile organic compounds with volatile fatty acids (VFAs) and thioalcohols being the primary components (Supplementary Information Figure S1)^8–10^. Thioalcohols, despite being present in trace amounts, are the most pungent voaltiles^9^. Natsch et al.^2,11^ identified trace amounts of four different thioalcohols in axillary secretions with 3-methyl-3-sulfanylhexan-1-ol (3M3SH) being the most abundant. 3M3SH is generated from the odourless precursor Cys-Gly-3M3SH, an l-cysteinylglycine dipeptide-conjugated alcohol that is secreted onto the surface of the skin by apocrine glands^12^. We and others have shown that a limited number of axillary staphylococcal species take up and metabolise Cys-Gly-3M3SH^3^. The precursor enters the cell through a proton-coupled oligopeptide di-/tripeptide transporter (DtpT) which is conserved across all staphylococcal species^13^, meaning it cannot be a unique requirement for thioalcohol production. The presumed biochemical pathway for Cys-Gly-3M3SH biotransformation following its uptake is sequential metabolism by a dipeptidase to release glycine and cleavage by a C-S β-lyase to liberate the volatile 3M3SH (Fig. 1A); to date, the enzymes involved have not been identified in odour producing staphylococci. Here, we describe the biochemical basis of thioalcohol formation through our identification and structural characterisation of a unique intracellular enzyme essential for 3M3SH liberation in the odour-forming species *Staphylococcus hominis*. Using a combination of structural biology and biochemistry, we demonstrate how this enzyme is substrate selective for the thioalcohol precursor Cys-3M3SH. This represents a new level of understanding on how specific microbes biochemically contribute to axillary malodour, an essential prerequisite for more targeted strategies to inhibit body odour.

## Results

### Discovery of a unique C-S lyase involved in the formation of body odour

By screening a range of axillary *Staphylococcus* species and strains, we identified those coagulase negative staphylococci (CoNS) able to take up and convert Cys-Gly-3M3SH to 3M3SH (Fig. 1B and Supplementary Information Figure S2A). Among these, a monophyletic group of CoNS emerged as the most efficient biotransfomers of Cys-Gly-3M3SH (Fig. 1B Group 1 [G1]), along with *Staphylococcus* species from two other distinct clades (Fig. 1B Groups 2 and 3 [G2&G3]). Strikingly, *Staphylococcus epidermidis*, the dominant staphylococcal species present on the skin including the axilla^14^, does not metabolise Cys-Gly-3M3SH (Fig. 1B, Supplementary Information Figure S2B and C), and nor do other species of human associated staphylococci such as *Staphylococcus capitis* and *Staphylococcus aureus* (Fig. 1B; Supplementary Information Figure S2C). The G1 clade contains *S. hominis*, a species which is strongly associated with body odour, along with *Staphylococcus lugdunensis* and *Staphylococcus haemolyticus*, which have been previously linked to thioalcohol production^3^. In order to elucidate the molecular basis for this highly limited phenotype in staphylococci, we searched staphylococcal genomes for enzymes likely to be involved in the generation of volatile thioalcohols. All staphylococcal genomes encode a DtpT orthologue, involved in precursor uptake^15^ and PepA, the likely peptidase required for removal of the glycine from the Cys-Gly-3M3SH (Fig. 1A, B), so we reasoned that the lyase step would be unique. Cleavage of Cys-3M3SH to produce 3M3SH involves a β-elimination from an amino acid substrate. As this type of chemistry is most commonly performed by enzymes containing pyridoxal phosphate (PLP), we focused our search on unusually distributed PLP-dependent enzymes (PLP-DEs) present in staphylococci^9^ including PLP-DEs from the Cys/Met metabolism family. All staphylococci contain orthologues of MetC, a cystathionine β-lyase that converts cystathionine to homocysteine as the penultimate step in methionine biosynthesis^16^. Previous work demonstrated that MetC from *S. haemolyticus*, a species in the G1 clade, does not catalyse Cys-3M3SH cleavage, suggesting another PLP-DE is responsible^17^. Orthologues of another PLP-DE identified in *Bacillus subtilis*, the putative aspartate transaminase PatA^18^ (Fig. 1B), are ubiquitously distributed in staphylococci. However, a second related protein, known as PatB in *B. subtilis*, is present in a small number of staphylococci only (Fig. 1B). In fact, the occurrence of a gene encoding this protein correlates precisely with the detection of Cys-Gly-3M3SH breakdown in our in vivo biotransformation assay (Fig. 1B). While the PatB enzymes are poorly characterised and the genes are not associated with amino acid metabolism gene clusters or operons^19^, the orthologues from *B. subtilis* (PatB) and *Escherichia coli* (MalY) are known to have cystathionine β-lyase activity^19,20^, suggesting that these enzymes might also be capable of Cys-3M3SH cleavage.

**Figure 2:**
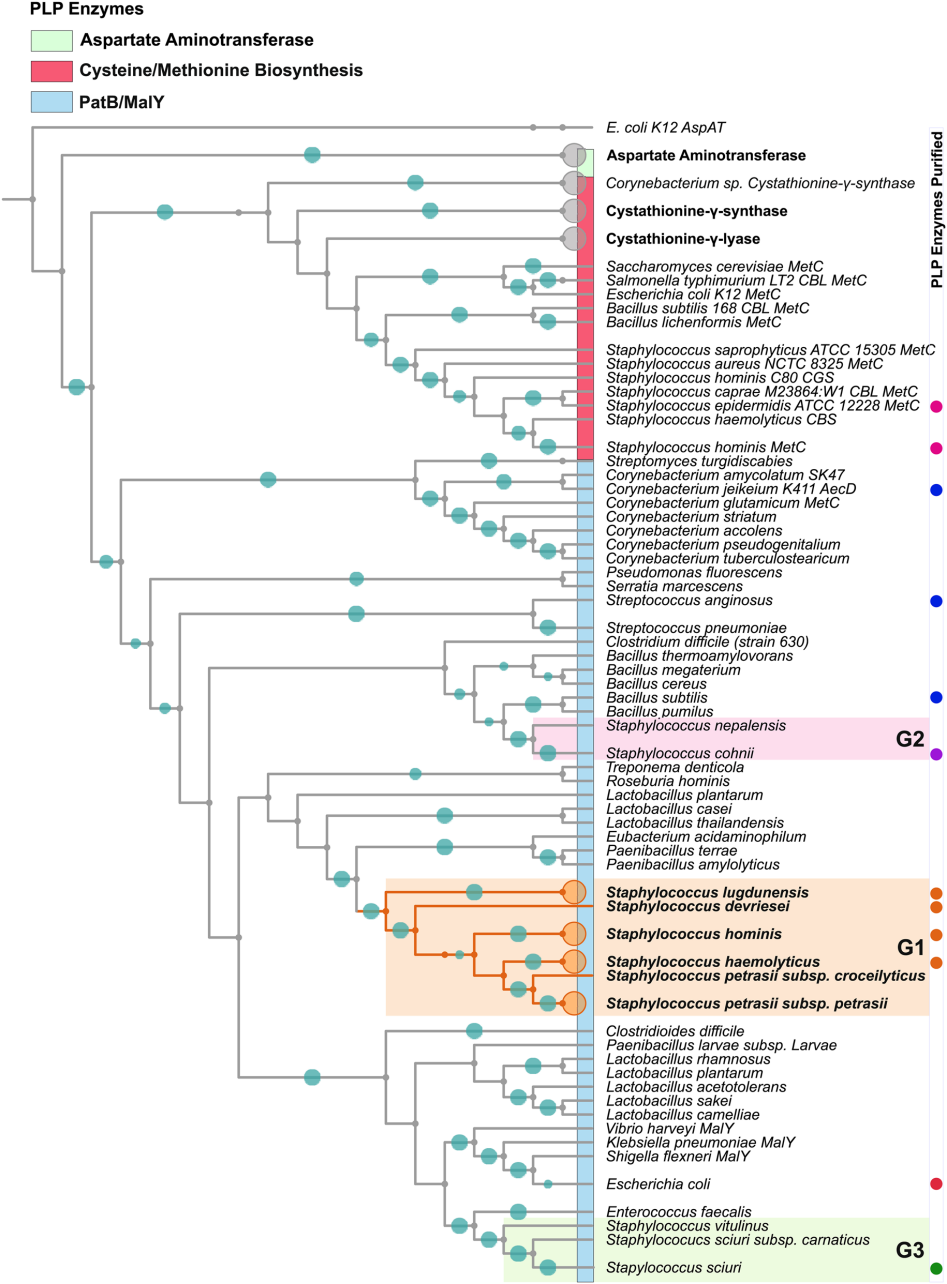
Malodour producing staphylococci contain a unique C-S lyase enzyme. Maximum likelihood tree of PLP dependent C-S lyases from representative bacterial species. A unique PLP dependent PatB enzyme is found in a distinct phylogenetic clade of staphylococcal species, which we refer to as malodour producing staphylococci (coloured orange, G1). Coloured dots represent selected PLP-dependent enzymes purified for further biochemical characterisation. Orthologous PatB enzymes found in other *Staphylococcusal* spp. are indicated by G1, G2 and G3. Phylogenetic tree was generated using iTOL (https://itol.embl.de/).

Mapping the few examples of staphylococcal PatB-like enzymes onto the global phylogeny of the *Staphylococcus* genus, suggests that horizontal gene transfers into staphylococcal lineages occurred on three independent occasions with the earliest being into an ancestor of G1, likely from a *Bacillus*-like ancestor (Fig. 2). This clade of PatB-containing staphylococci includes *S. hominis*, a strong producer of thioalcohol-based malodour, and its signature enzyme, which we named ShPatB, was thus studied further. Core genome analysis reveals ShPatB is a conserved core gene present in *S. hominis* (Supplementary Information Figure S3). To test whether ShPatB is important for malodour production, we expressed the gene in a non-malodour producing strain of *S. aureus* and were able to measure 3M3SH production in vivo (Supplementary Information Figure S2 D). This demonstrates that ShPatB is both necessary and sufficient for thioalcohol-based odour production in the human underarm (Fig. 1A, B).

### ShPatB is selective for branched aliphatic thioalcohol ligands

**Figure 3:**
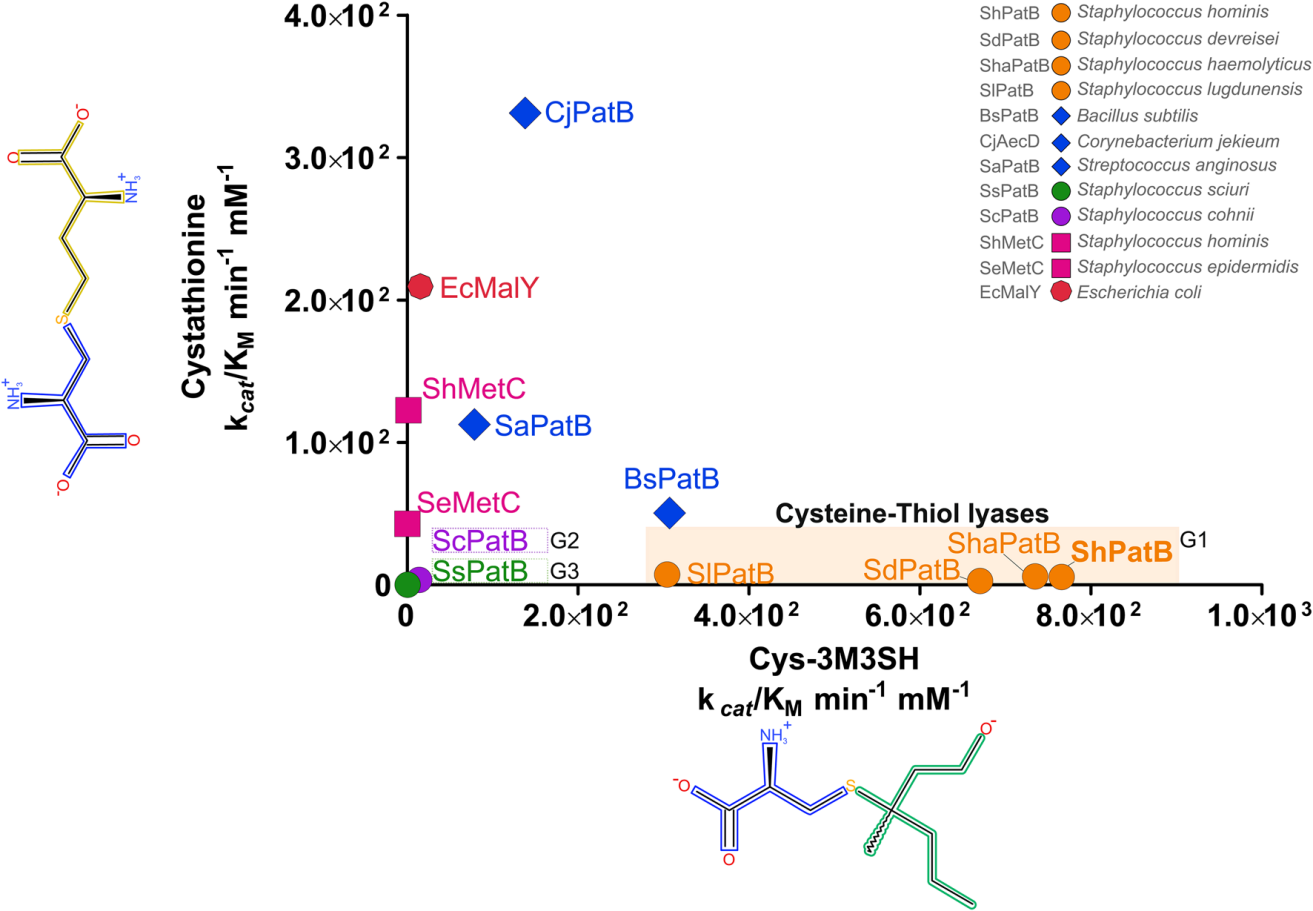
Malodour producing staphylococci PatB enzymes are selective for Cys-3M3SH. Comparison of catalytic efficiencies (K_cat_/K_M_) for selected PLP dependent C-S lyases against classical C-S lyase substrate l-cystathionine (y-axis) and l-Cys-3M3SH (x-axis). We show distinct clusters of activity across the PLP-DEs. MetC enzymes are selective for cystathionine only while G1 malodour-producing staphylococci are substrate selective for Cys-3M3SH, we now classify these enzymes as cysteine-thiol lyases. Other PatB orthologs (blue) display activity across both substrates whereas orthologous PatB enzymes from staphylococci (G2 & G3) show little or no activity with either substrates. Groups of staphylococcal PatB enzymes are highlighted.

Next, we cloned and overexpressed genes encoding a representative range of PatB-type enzymes, with two MetC proteins as controls (Fig. 2, denoted by coloured dots) and purified the proteins for biochemical analysis. We compared the catalytic efficiencies (k_*cat*_/K_M_) of these 12 PLP-DEs for the malodour substrate Cys-3M3SH and the classical C-S lyase substrate cystathionine (Fig. 3). We observe distinct clusters of catalytic activity, with G1 PatB enzymes showing higher activity against the malodour substrate Cys-3M3SH compared to all the other PLP-DEs (Fig. 3). In particular, ShPatB exhibits selectivity towards Cys-3M3SH with a catalytic efficiency 138-fold higher that that towards cystathionine (766 M^−1^ min^−1^ and 5.53 M^−1^ min^−1^, respectively) (Fig. 3, Supplementary Information Figure S4, Table S1 and Table S2). The staphylococcal PatB-like enzymes from G2 and G3 species show very low activity towards Cys-3M3SH, suggesting that they do not contribute significantly to body odour formation given the likely micromolar concentrations of precursor present in the axilla (Fig. 3). The PatB enzymes from non-axillary microbes *B. subtilis* (BsPatB) and *Streptococcus anginosus* (SaPatB) did not discriminate between the two ligands (Fig. 3), while the enzymes from *Corynebacterium jeikeium* (CjAecD) and *E. coli* MalY (EcMalY) had higher activities against cystathionine, similar to the MetC enzymes included (ShMetC and SeMetC), which have little or no activity against Cys-3M3SH (Fig. 3 and Supplementary Information Figure S4). We also measured enzyme activity against Felinine, a close structural analog of Cys-3M3SH and a putative pheromone precursor found in cat urine^21^, and observed a very similar activity profile to that seen for Cys-3M3SH (Supplementary Information Figure S4, Table S3). Compared to cystathionine, the malodour precursors differ significantly in the side chains attached to the cysteine thiol, these being branched and hydrophobic, rather than linear with ionisable amino and carboxylate groups which are expected to be charged at physiological pH (Supplementary Information Figure S4). As ShPatB and G1 PatB enzymes have novel selectivity for cysteine-conjugated thioalcohol ligands, we propose that these enzymes are cysteine-thiol lyases (C-T lyases) distinct from C-S lyases acting on a broad range of cysteine-conjugated ligands (such as BsPatB and CjAecD).

### Structural characterisation reveals a hydrophobic thioalcohol binding pocket

To explore the structural basis of ShPatB selectivity for these more hydrophobic malodour precursors compared to the broader C-S lyase substrate activity of BsPatB, crystal structures of the two proteins were solved and refined to resolutions of 1.6 Å (PDB ID 6QP2) and 2.3 Å (PDB ID 6QP3) respectively (Supplementary Information Figure S5A, B). ShPatB and BsPatB are homodimeric with each subunit containing a PLP moiety covalently bound to a conserved lysine residue in the catalytic site (Lys246 and Lys234 in ShPatB and BsPatB, respectively) in what is termed the internal aldimine state (Supplementary Information Figure S5 A and B). Overall, ShPatB and BsPatB are structurally conserved and belong to the type onefold of PLP-DEs^22^ (Supplementary Information Figure S5D). Absorption spectra indicate the presence of PLP (410 nm) covalently bound to ShPatB (Supplementary Information Figure S6A). We note, upon addition of Cys-3M3SH and additional peak at ~ 500 nm concomitant with a decrease at 410 nm (Supplementary Information Figure S6B), this species is most likely the external aldimine intermediate. A peak in this range typically indicates the presence of a PLP intermediate and is observed in cystathionine β-lyases^23^. After 30 s the peak reduces with a slight increase at 410 nm. As ShPatB is a β-C-S lyase we do not see any activity with l-methionine which is a γ-lyase substrate (Supplementary Information Figure S6C).

**Figure 4:**
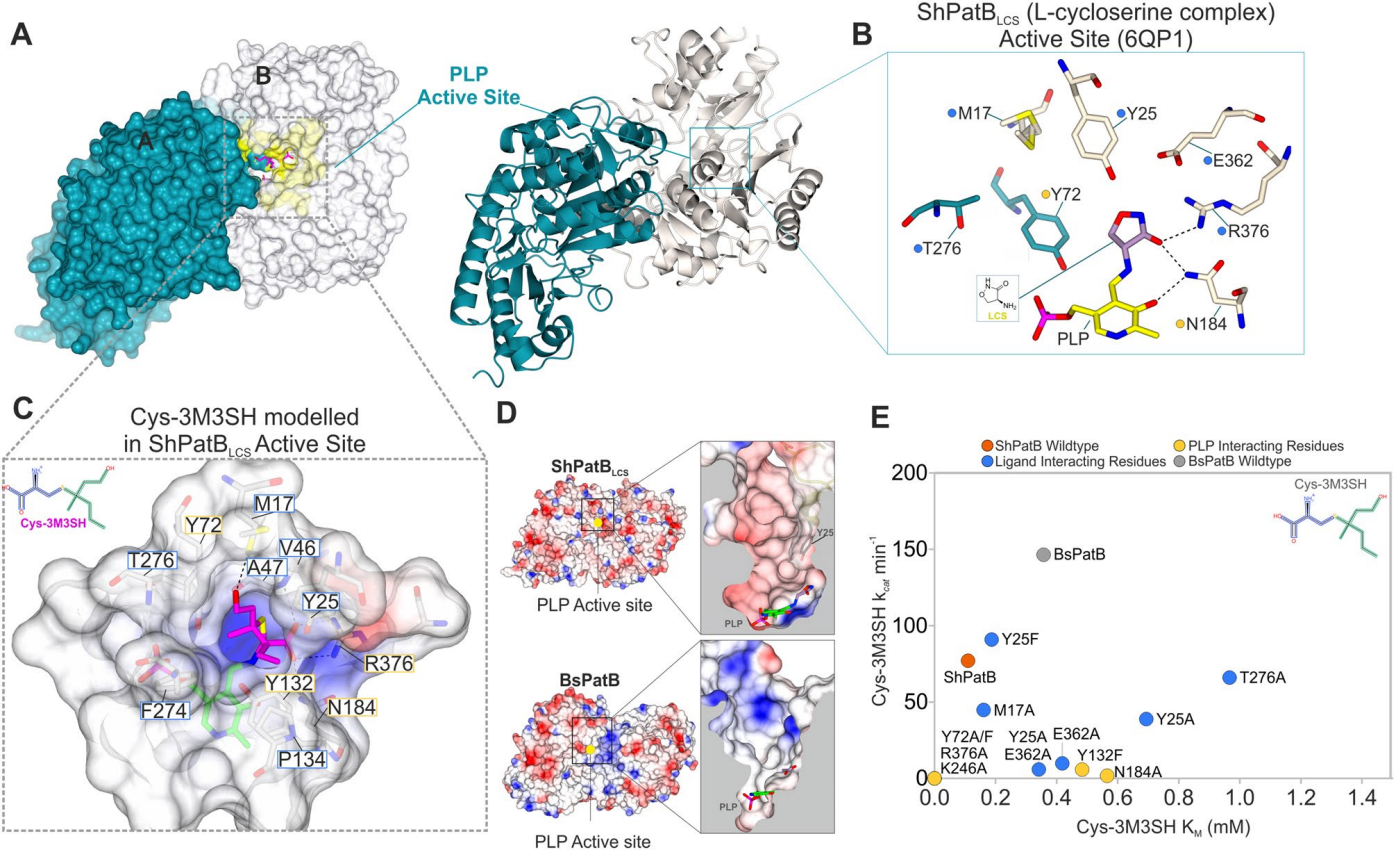
Structural characterisation of ShPatB binding site. (**A**) Homodimeric structure of ShPatB. Both surface and ribbon representation are shown. (**B**) Zoomed view of ShPatB bound in complex with cycloserine (PDB ID 6QP1). l-cycloserine is shown in the external aldimine form bound to PLP. Coloured residues Y72 T276 denote chain A while all other residues are from chain B. (**C**) Modelled Cys-3M3SH complex in ShPatB_LCS_ structure. The Cys-3M3SH ligand is modelled in the external aldimine form and docked onto the ShPatB_LCS_ structure. Cys-3M3SH is coordinated by conserved ion pairing of its carboxylate group with the side chain of Arg376. (**D**) Electrostatic surface potential for ShPatB_LCS_ and BsPatB respectively. Zoomed in views of the active site indicating the possible role of Y25 in mediating apolar interactions. In the 90 °C rotated view, we clearly see a narrow hydrophobic pocket in wild-type ShPatB whereas BsPatB lacking Y25 appears to have a more solvent accessible exposed binding site. (**E**) In vitro kinetics of ShPatB mutants. Mutagenesis highlights the importance of the conserved PLP interacting and ligand binding residues while revealing the importance of Y25 and E362. All structural images were generated in CCP4MG (https://www.ccp4.ac.uk/MG/).

**Figure 5:**
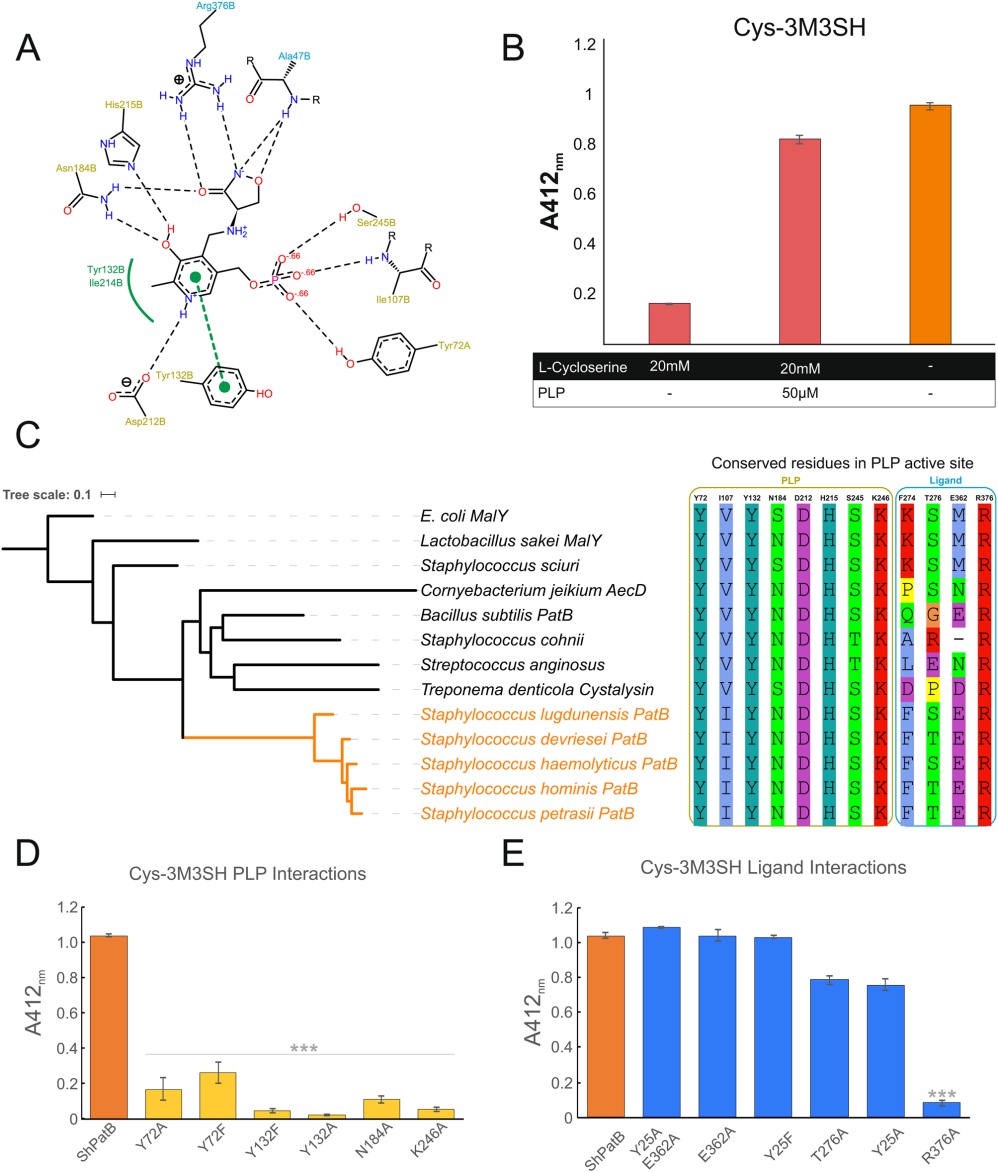
Functional analysis of ShPatB active site variants. (**A**) Schematic diagram of ShPatB + cycloserine complex. PLP interacting residues are indicated in yellow and ligand interacting residues are in blue. (**B**) Cycloserine in vitro inhibition of ShPatB with Cys-3M3SH as the substrate. End point in vitro biotransformation assay of ShPatB (2.5 μM) incubated with Cys-3M3SH (2.5 mM) in the presence or absence of cycloserine. Release of 3M3SH was labelled with DTNB and absorbance measured at 412 nm (y-axis). Cycloserine was thought to bind irreversibly to PLP^27^ to inactivate ShPatB however, we show that inhibition is reversed by excess PLP thus regenerating ShPatB for catalysis of Cys-3M3SH. (**C**) Multiple sequence alignment of C-S-β-lyases showing conserved PLP and ligand interacting residues. (**D**) In vivo biotransformation of Cys-3M3SH with ShPatB binding site PLP mutants and (**E**) ShPatB ligand interacting residues. Phylogenetic tree was generated using iTOL (https://itol.embl.de/).

In the course of a typical PLP-DE catalysed reaction, upon substrate binding, the ε-amino group of the amino acid substrate displaces the lysine residue from the PLP to form an external aldimine^24–26^ (Supplementary Information Figure S8). The PLP and the α-amino group of the displaced lysine next facilitate electron pair and proton shuttling that lead to breakage of the C-S bond and release of 3M3SH (see Supplementary Information Figure S8 for suggested mechanism). We made several attempts to crystallise ShPatB and subsequent ShPatB catalytic mutants in the presence of the ligand Cys-3M3SH but were unable to obtain crystals suitable for X-ray structure determination. As we were unable to capture reaction intermediates by soaking ShPatB or BsPatB crystals with Cys-3M3SH, we sought insight into the mode of substrate binding by growing crystals of ShPatB in the presence of l-cycloserine (LCS). LCS is a known PLP-DE inhibitor^27^, that forms an external aldimine complex with PLP thereby inhibiting ShPatB (Fig. 5A, B). The structure solved at 1.4 Å (PDB ID: 6QP1) confirms the formation of the external aldimine and reveals the LCS and PLP interacting residues in the binding pocket of ShPatB (Fig. 4A, B, D and Supplementary Information Figure S5C). Mutation of key conserved PLP and LCS interacting residues reduced activity both in vitro and in vivo (Figs. 4E and 5D, E), demonstrating their important roles in binding and catalysis^24^. Supplementary Information Table S4 summarises the steady state kinetics for all ShPatB mutants analysed for Cys-3M3SH in vitro biotransformation. Common to PLP-DEs, a highly conserved arginine residue (Figs. 4B and 5A, C) forms an ion-pairing interaction with the carboxylate group of the amino acid moiety of the various amino acid substrates^22,28,29^. In ShPatB, we infer this arginine to be Arg376 (Supplementary Information Figure S5A); in the structure of the inhibitor complex, it forms a polar interaction with the C=O of LCS and is well-positioned to form a salt-bridge with the Cys-3M3SH adduct. Moreover, substitution of this residue with alanine abolishes activity both in vitro and in vivo (Figs. 4E and 5E, respectively).

**Figure 6:**
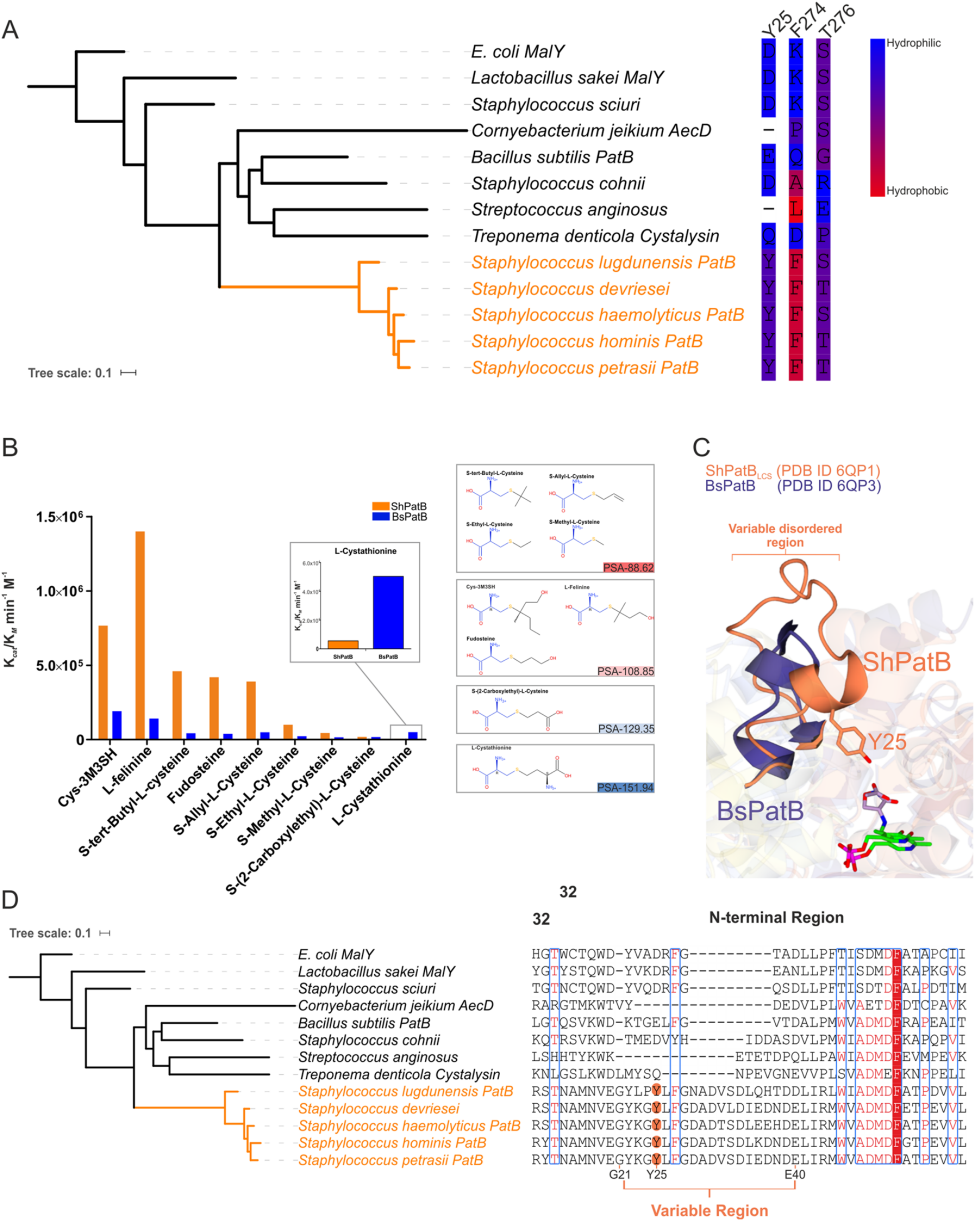
ShPatB is selective for aliphatic cysteine-S-conjugates. (**A**) ShPatB and other G1 PatB enzymes contain unique hydrophobic residues. (**B**) Catalytic efficiencies (K_*cat*_/K_M_) show that ShPatB has significantly higher activity for aliphatic cysteine-S-conjugate substrates compared to BsPatB. We hypothesise that unique hydrophobic residues (Tyr25 and Phe274) found only in malodour producing staphylococci mediates apolar contacts hence increased selectivity of ShPatB for these substrates. PSA indicates polar surface area calculated by BIOVA Draw 2018. (**C**) Structural comparison of ShPatB N-terminal region with BsPatB. BsPatB lacks equivalent Tyr25 found in ShPatB and does not provide a hydrophobic pocket. (**D**) ShPatB contains a highly variable region located at N-terminus. Structural sequence alignments shows a highly divergent N-terminal region between malodour producing staphylococci and orthologs. Y25 is coloured orange in malodour producing staphylococci. Red background indicates strictly conserved, red text—highly similar. The alignment was generated using MAFFT-LiNS in Jalview (https://www.jalview.org/) the graphic was prepared on the ESPript 3.0 server (https://espript.ibcp.fr/ESPript/cgi-bin/ESPript.cgi). Phylogenetic trees were generated using iTOL (https://itol.embl.de/).

While the reaction mechanism of PLP-DEs action is well established, determining the substrate specificity of novel PLP-DEs remains a major challenge^24^. To gain insights into ShPatB selectivity we modelled the structure of the external aldimine form of the enzyme bound to Cys-3M3SH. The PLP adduct of Cys-3M3SH was superimposed onto equivalent atoms of the LCS external aldimine bound to ShPatB so that the α-carboxylate of the substrate forms the conserved ion-pairing interaction with Arg376. In this conformation, the side chain of Cys-3M3SH projects from the deeply recessed PLP binding pocket towards the protein surface (Fig. 4C and Supplementary Information Figure S7A and B). The aliphatic 3M3SH species fits, with minimal steric hindrance, into a spacious apolar pocket formed by the side chains of Tyr25, Val46, *Tyr72*, Val108, Pro134,*Phe274* and *Thr276* (where italics denotes a residue from the partner subunit of the dimer) (Fig. 4C). The hydrophobic character of this pocket provides few, if any, polar groups to form interactions with the side chain of cystathionine which would be expected to be zwitterionic at physiological pH (Fig. 4C, D). As a result, cystathionine binding would be accompanied by the development of unsolvated, or poorly solvated charge, lowering affinity and accounting for discrimination against this substrate as observed in our kinetic data (Fig. 3). We suggest that the hydrophobic character of this pocket accounts for the selectivity toward malodour substrates. To test this, we measured the kinetics of the ShPatB catalysed reaction with a range of cysteine-S conjugate ligands that varied in side chain length, the presence or absence of side chain branching, and side chain polarity. ShPatB clearly prefers branched aliphatic side chains followed by linear hydrophobic side groups while excluding linear charged ligands (Fig. 6B). This structural accommodation of malodour substrates represents a unique evolutionary trajectory for ShPatB not seen in other reported PatB enzymes to date.

To gain insights into this constrained substrate binding site in ShPatB, we examined a unique tyrosine residue (Tyr25) that extends into the active site in each subunit with the phenolic side chain projecting towards the PLP moiety (Figs. 4A and 6C). Tyr25 is part of a sequence divergent N-terminal region, and this residue is found only in malodour producing staphylococci (Fig. 6D). This N-terminal region is often unresolved in PLP-DE structures, and indeed, we could resolve this region only in the ShPatB_LCS_ structure. The introduction of a Tyr25Ala substitution resulted in a sixfold increase in K_M_, significantly affecting Cys-3M3SH binding compared to wild-type ShPatB (Fig. 4E). We hypothesise that Tyr25 contributes to a specific hydrophobic surface in ShPatB, absent in BsPatB and other PLP-DEs (Figs. 4C and 6C), that efficiently orientates the 3M3SH moiety. In contrast to ShPatB, the BsPatB binding cavity is more solvent exposed and composed of charged residues (Fig. 4D), thus enabling the binding of polar substrates like cystathionine and the hydroxyl group of the 3M3SH moiety of Cys-3M3SH.

Supporting this, the substitution of Tyr25 to a similarly hydrophobic phenylalanine (Tyr25Phe) does not significantly affect Cys-3M3SH binding (Fig. 4E). The shape of the hydrophobic pocket is critical for Cys-3M3SH binding; mutating Thr276 to Ala resulted in an 8.5-fold increase in ShPatB K_M_ for Cys-3M3SH (Fig. 4E). Within the neighbourhood of this apolar pocket, we observe hydrophobic residues (Tyr25, Phe274) that are unique to malodour producing staphylococci (Fig. 6A). Taken together, our observations suggest that the hydrophobic binding site in ShPatB is a key determinant of this enzyme's selectivity towards malodour-producing substrates.

### Evolutionary phylogeny of malodour producing staphylococci

**Figure 7:**
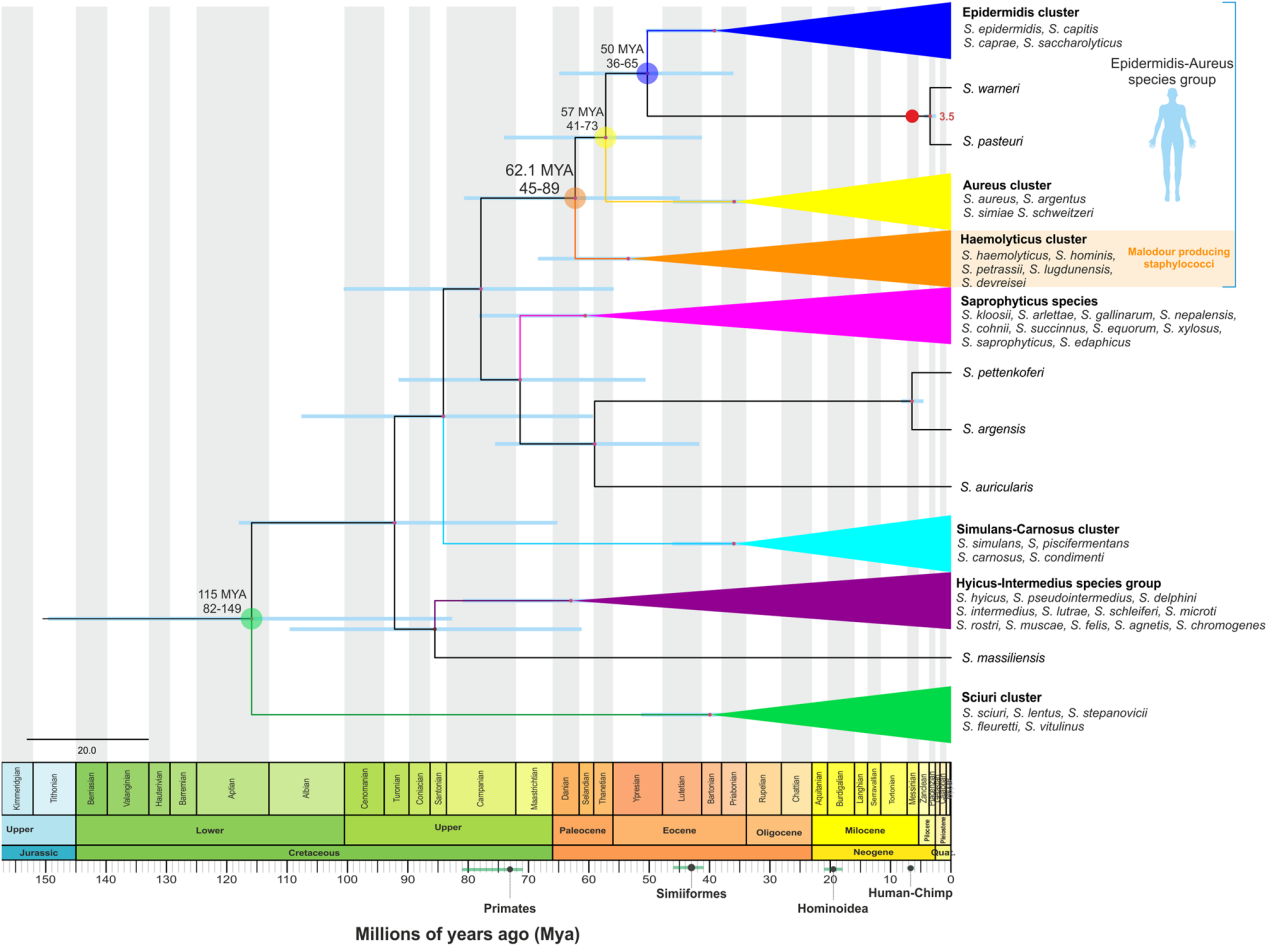
Divergence time and evolution of *Staphylococcus* spp. Bayesian maximum clade credibility tree for representative *Staphylococcus* spp. based on core genome sequences. Branch lengths are proportional to divergence times (millions of years ago, MYA). Blue bars represent 95% highest posterior density of node age. Our data show the diversification of malodour producing staphylococci approximately 60 MYA. Phylogenetic tree was generated using FigTree V1.4.4 (https://tree.bio.ed.ac.uk/software/figtree/).

As noted previously (Fig. 2), the distribution of PatB enzymes among staphylococci is limited to a handful of species. In contrast, they have a much broader distribution across the *Bacillus* genus suggesting an ancient horizontal gene transfer (HGT) event into staphylococci. From our phylogenetic analysis, we infer this happened at least three times in staphylococci (Fig. 2), although only one of these events occurred in human associated staphylococci and led to an enzyme with high activity against Cys-3M3SH with counter selectivity against cystathionine (the G1 PatB enzymes). As the G1 enzymes are present in species that form a clear monophyletic group of staphylococci, we attempted to date the split of this clade from the other non-odour producing staphylococci to age the process of thioalcohol production. In order to determine the evolutionary phylogeny, we generated a core genome alignment of representative *Staphylococcus* sp. (1B). This core genome alignment was used to infer a time-scaled evolutionary phylogeny of *Staphylococcus* species. We used Bayesian analysis to estimate *Staphylococcus* species divergence time (Supplementary Information Figure S9). For the temporal scale, we used the divergence time between *Staphylococcus warneri* and *Staphylococcus pasteuri* estimated from the TimeTree database^30^. We show the appearance and diversification of malodour producing staphylococci from the most recent common ancestor (MRCA) approximately 60 million years ago (MYA) (95% highest posterior density (HPD) 45–89 MYA) (Fig. 7). This would imply that the emergence of this process in the staphylococcal population occurred around the same time as the early divergence of primates and the appearance of the suborder Haplorhini^31^. While several studies have characterised the human skin microbiome (reviewed by Grice and Segre^32^) relatively little is known about the composition of non-human mammals, especially using next-generation sequencing technologies. Humans have a distinct axillary microbiota that is typically less diverse compared to other primates. However, Council et al.^33^ showed that, in the absence of antiperspirant or deodorant usage, humans share a similar axillary microbiome to apes. They identified a core axillary microbiome dominated by *Corynebacterium* along with *Anaerococcus*, *Prevotella* and *Staphylococcus* as the most abundant taxa. While there is certainly error in this estimate of 60 MYA, we believe that the most parsimonious explanation is that this malodour producing group of staphylococci was associated with the ancestral populations of humans going back towards the divergence of primates.

## Discussion

Human body odour is produced by the bacterial metabolism of odourless secretions from the apocrine gland. The role of the axillary microbiota in the formation body odour has long been established^1,9^; however, the molecular basis of malodour pathways from resident axillary microbes has not been fully elucidated. Thioalcohols, along with VFAs, are the primary components of axillary malodour^8,9^. Until now, the identification of the enzyme responsible for the C-S cleavage of thioalcohol precursors such as Cys-3M3SH was unknown. Initially, the C-S lyase activity was attributed to *Corynebacterium* species^2,9^; however, Bawdon et al.^3^ later demonstrated that *S. hominis*, *S. lugdunensis* and *S. haemolyticus*, but not *Corynebacterium* species, metabolised the physiological dipeptide malodour precursor Cys-Gly-3M3SH. Here, we show these *Staphylococcus* species form a distinct monophyletic group; we also show that other *Staphylococcus* species present within this group are capable of thioalcohol production, which was previously unknown (Supplementary Information Figure S2C). This is in agreement with Starkemann et al.^34^ who identified *S. haemolyticus* as an efficient biotransformer of Cys-Gly-3M3SH in contrast to other axillary species including *S. epidermidis* and corynebacteria. Initial attempts to identify the C-S lyase pinpointed several MetC- and MalY-type enzymes, although Troccaz et al.^17^ clearly demonstrated that staphylococcal MetC enzymes are not involved in the formation of volatile sulphur compounds. Subsequently, the MalY-type C-S lyase AecD from corynebacteria was associated with precursor cleavage and subsequent thioalcohol-based odour formation^9^.

In this study, we identify PatB in *S. hominis* as the C-S lyase responsible for thioalcohol liberation from Cys-3M3SH. To date, this is the first structural characterisation of the thiol-based axillary malodour pathway in *S. hominis*. ShPatB is a MalY-type enzyme that is uniquely distributed among staphylococci; we observed orthologs present in three groups of *Staphylococcus* species (clades G1, G2 G3). We kinetically characterised these PatB C-S lyases from staphylococci in vitro, alongside MetC-type and other MalY-type PatB PLP-DEs with previously reported C-S lyase activity. Our results demonstrate the PatB enzymes from G1 staphylococci are selective for the malodour substrate Cys-3M3SH over the typical C-S lyase substrate cystathionine, compared to other PatB enzymes. ShPatB has the highest catalytic efficiency for Cys-3M3SH, 5.5 fold higher than CjAecD from *C. jeikeium*. We also showed that CjAecD is selective for cystathionine over Cys-3M3SH, in agreement with its role in methionine biosynthesis in *Corynebacterium* species^35^. Given that corynebacteria cannot metabolise the dipeptide thiol precursor in vivo, their role in thioalcohol production in the axilla would appear to be limited.

The structure of ShPatB provided significant insights into how this enzyme has evolved selectivity for the thiol precursor Cys-3M3SH. ShPatB is a PLP-DE, with a core active site that is structurally conserved across all C-S lyases (Supplementary Information Figure S5C); however, ShPatB contains a hydrophobic pocket comprising unique residues not found in any other PatB enzymes other than malodour producing staphylococci (Fig. 6). This hydrophobic pocket in ShPatB is essential for Cys-3M3SH selectivity. Our phylogenetic analyses suggest ShPatB may have evolved from a common ancestor (Fig. 2). Despite sharing high structural similarity to closely related orthologs, we observed significant differences in substrate activity among these MalY-type PatB enzymes. In contrast to the roles of other MalY-type PatB enzymes as redundant MetC replacements in sulphur-containing amino acid metabolism^18,23,35^, ShPatB is selective for aliphatic cysteine-S-conjugates only, while discriminating against polar ligands such as cystathionine.

The composition of the modern human axilla is considered unique in composition and diversity relative to other primates^33^. The increasing abundance of *Staphylococcus* species^6,32^ can be considered indicative of an evolutionary trajectory toward modern humans. The abundance of staphylococci in the axilla is dominated by *S. epidermidis*^5^; indeed, the phylogeny of S*taphylococcus* species suggests that the human associated species *S. epidermidis* and *S. aureus* are the most recently diversified, based on our analysis (Fig. 7), while the appearance and diversification of malodour producing staphylococci occurs approximately 60 MYA. While we recognise the error in these estimates, we propose that the most parsimonious explanation is that this group of malodour producing staphylococci must have been present in ancestral human populations potentially dating back to primates. Based on this assumption, we suggest that thioalcohol production predates the emergence of *Homo sapiens* as a species.

This discovery raises important questions about the role of odour production in the evolution of modern humans. The emergence of an enzyme present in bacteria found in the human underarm with unique activity to catalyse production of 3M3SH suggests selection pressure for the production of odours over an extended period of evolutionary time. This was presumably driven by an evolutionary advantage for both the host (primate, human), that actively produces the odour precursors for no other apparent physiological reason, and their microbiota, which converts them to volatile odorous molecules. Here, we have identified a definite substrate-product relationship, namely the conversion of specific thioalcohol precursors by malodour producing staphylococci. We show that *S. epidermidis* unequivocally does not metabolise these precursors, despite being the most abundant *Staphylococcus* species present in the axilla. Could these thioalcohol precursors secreted by the apocrine sweat glands be significant for the ecological success of *S. hominis* in the human axilla? This raises important and as yet unanswered questions regarding the mechanisms that govern the structure and composition of the axillary microbiome.

## Methods

### Bacterial strains, media, reagents, chemicals and plasmids

All bacterial strains and plasmids used in this study are listed in Supplementary Information Table S6 and Table S7 respectively. Most of the *Staphylococcus* strains were isolated during a Unilever human axillary malodour project. Dr Malcom Horsburgh (University of Liverpool) kindly provided a number of *Staphylococcus* strains. All standard chemcials and buffers were purchased from Sigma-Aldrich. DTNB, PLP and S-Benzyl-l-cysteine were purchased from Sigma-Aldrich. Physiological malodour substrates Cys-Gly-3M3SH and Cys-3M3SH were custom synthesised by Concept Life Sciences. Stock concentrations were prepared at 10 mM in M9 buffer.

### Cloning, expression and purification of C-S lyase proteins

C-S lyase proteins for purification were cloned into the expression vector pBADcLIC with a C-terminal Hisx10 tag under the control of arabinose. Full length proteins were either amplified by PCR or synthesised as gBlock (IDT) fragments. For PCR amplification, full-length genes were amplified by PCR from the host organism with additional overhangs for cloning (final recombinant protein MGGGFA <INSERT> ENLYFQGHHHHHHHHHH*). PCR fragments were purified and cloned into pBADcLIC by standard ligation-independent cloning (LIC). Oligonucleotides were purchased from Sigma-Aldrich. For protein expression, all pBAD plasmids were transformed into *E. coli* MC1061. For overexpression of C-S lyases proteins, overnight cultures of *E. coli* MC1061 harbouring a pBAD plasmid expressing a C-S lyase were grown in LB at 37 °C, 200 rpm. Overnight cultures were diluted to an A600_nm_ 0.05 in 1-L LB supplemented with 100 mg mL^−1^ ampicillin. Cultures were incubated at 37 °C, 200 rpm to OD600nm 0.4–0.5 and induced by addition of 0.01% arabinose. After 8 h expression, cells were harvested by centrifugation at 5,000 g for 15 min, resuspended in 35 mL resuspension buffer (50 mM Potassium Phosphate (KPi), 20% glycerol, 200 mM NaCl and 10 mL imidazole, pH 7.8), and stored at -80 °C. All subsequent steps were carried out at 4 °C. Resuspended cells were thawed and supplemented with 1 mM AEBSF protease inhibitor (Thermo Fisher Scientific). Cells were lysed by sonication (3 s pulse, 7 s pause – 3 min). Lysates were clarified by centrifugation at 27,000*g* for 30 min. The clarified supernatant was loaded onto a 5 mL HisTrap column (GE Healthcare) and affinity purified on an AKTA Start (GE Healthcare) using a standard protocol as per manufacturer’s instructions. Fractions were pooled and concentrated to 15–20 mg mL^−1^ for crystallisation or biochemical kinetic assays. Proteins were stored in 50 mM KPi and 200 mM NaCl. For crystallography experiments proteins were stored in 20 mM Tris, pH8 and 50 mm NaCl.

### ShPatB site-directed mutagenesis

Site directed mutagenesis was used to generate variants of *S. hominis* PatB for kinetic analyses. Native ShPatB cloned into pBADcLIC was used to individually generate targeted mutants. To introduce the mutation, high fidelity inverse PCR was performed using divergent primers with one per pair being mutagenic. Mutagenic PCR products were circularised by blunt end ligation. All mutants were verified by Sanger sequencing (GATC Biotech). ShPatB variants were transformed into *E. coli* MC1061 for protein expression and into *E. coli* BW25113 for in vivo kinetic analyses.

### In vivo Cys-Gly-3M3SH biotransformation assays

Resting cells were used for in vivo biotransformation of the malodour precursor Cys-Gly-3M3SH. Overnight cultures were harvested by centrifugation at 3,000*g* for 10 min and resuspended in sterile M9 buffer. Cells to an OD600_nm_ of 5 were added to 2.5 mM substrate and M9 to a final volume of 200 μL. Reactions were incubated at 37 °C for 5 h. Liberated thiols were quantified by dithionitrobenzoic acid (DTNB) labelling (see below). 100μL from each reaction was centrifuged at 15,000*g* for 2 min, 50 μL of the supernatant was added to 50 mM Tris–HCL (pH 8.0), 0.4 mM DTNB in a final volume of 200 μL. Reactions were measured at A412 nm in a Jenway 6305 spectrophotometer.

### Steady state in vitro kinetics

In vitro activity assays were performed using an Epoch2 (BioTek) plate reader. Reactions were measured in 96-well plate. A continuous assay, described previously^3^ was used to measure 3M3SH. A 200 μL reaction contained 0.25 mM enzyme, variable amounts of substrate and 0.4 mM DTNB in 50 mM KPi and 200 mM NaCl, pH 7.8. Cys-3M3SH substrate concentrations ranged from 2 to 7.8 mM. Each reaction was incubated at 37 °C for 30 min. Release of the thiol 3M3SH was measured by reaction with DTNB to form 2-nitro-5-thiobenzoic acid (TNB) which is measured at A_412_ nm every minute. Background rate of DTNB decomposition was subtracted in each assay. Michaelis–Menten plots were derived from 6 to 8 substrate concentrations using Prism Version 5.01 (GraphPad) to calculate the K*cat* and K_M_. Enzyme concentrations were assumed to be the concentration of PLP-bound enzyme with two active sites per enzyme. Velocity curves for all in vitro kinetics are shown in the supplementary information (Supplementary Information Figures S10–S12).

### Absorption spectroscopic analysis

The absorption spectrum between 300 and 600 nm of 25 μM purified ShPatB was initially determined at 10 nm intervals using a UV-transparent cuvette and the Epoch2 microplate spectrophotometer (BioTek). 5 mM of Cys-3M3SH or the negative control, l-methionine, was then added to the cuvette. The absorption spectra were then collected again at 15 s intervals.

### Crystallisation of ShPatB and BsPatB

Proteins were at 15 mg/mL in 20 mM Tris, pH8 and 50 mm NaCl. Crystals were grown in hanging drops (1:1 ratio) over 1 mL of the crystallisation solution (4% Tacsimate, pH 6 (Hampton Research) and 12% PEG 3,350) at room temperature in Corning Costar non-treated 24 well plates.

The condition was first identified using PEG/ION HT (Hampton Research) in 96 well MRC crystallisation plates. Shards of ShPatB crystals appeared overnight and were optimised in the 24 well plate format. ShPatB was co-crystallised with LCS. 20 mM l-cycloserine was added to the crystallisation solution for ShPatB_LCS_. Selected crystals were cryoprotected in 4% Tacsimate, pH 6, 12% PEG 3,350 and 20% glycerol. BsPatB was crystallised in a sitting drop (1:1 ratio) with 0.2 M Ammonium acetate, pH 7.1 and 20% PEG 3,350. Collected crystals of BsPatB were cryoprotected in 0.2 M Ammonium acetate, pH 7.1, 20% PEG 3,350 and 20% Glycerol.

### Data collection and structure determination

X-ray diffraction data were collected at the Diamond Light Source, UK on beamline i03 for ShPatB and ShPatBLCS and beamline i04 for BsPatB. The data collected for ShPatB, ShPatBLCS and BsPatB were indexed and scaled using the XDS pipeline on the xia2. All data reductions were performed using AIMLESS 0.729 30. Molecular replacement was used to obtain initial phase information for all structurally characterised proteins in this study using the structure of a *Clostridium difficile* aminotransferase (PDB ID: 4DQ6) on MOLREP^36,37^. The respective structures were refined using REFMAC5^38^. Refined coordinate sets and structure factors were deposited into the PDB with the entry codes 6QP2 for ShPatB, 6QP1 for ShPatBLCS and 6QP3 for BsPatB. Data collection statistics are provided in Supplementary Information Table S5.

### Cys-3M3SH ligand modelling

To describe how Cys-3M3SH could be coordinated, a structure of ShPatB docked with Cys-3M3SH in the l-cysteine form was created by rigid-body refinement. The atomic model and geometry dictionary for Cys-3M3SH were created in AceDRG^39^ which were then used to dock the ligand onto the cycloserine bound structure of ShPatB (PDB ID: 6QP1) in Coot^40^. The amino group of Cys-3M3SH was coordinated and anchored to atom N of the external aldimine, while the carboxyl group was coordinated to interact with Arg376. Using the restraints available in the ligand dictionary, the rest of the ligand was coordinated to fit in the remaining space of the likely binding pocket while keeping the stereochemistry of the ligand and ensuring minimal clashes with the protein structure.

### Phylogenetic analyses of PLP-dependent C-S lyases

To search for ShPatB homologs, its sequence (NCBI accession number WP_119633472.1) was used as the query for BLASTp (https://www.ncbi.nlm.nih.gov/BLAST/). We manually set a threshold of 30% sequence identity. Sequences were downloaded and aligned by MAFFT L-INS-i using Jalview. Phylogenetic analysis was performed by IQ-TREE. Phylogenetic tree was inferred by maximum likelihood with automatic model selection to find the best-fit model. Ultrafast bootstrap approximation was used give branch support. Phylogenetic trees were generated in iTOL (https://itol.embl.de/).

### *Staphylococcus* spp. core genome analysis

53 representative strains of *Staphylococcus* sp. were downloaded from NCBI Assembly (Supplementary Information Table S8). The genome assemblies were annotated with PROKKA and provided as input to Roary. Roary was run using default parameters except for the following: -e -n (to produce alignments with MAFFT) and -i 80 (lower amino acid identity than the default). Maximum likelihood phylogeny was performed using RAxML. A general time-reversible nucleotide substitution model was used, with gamma-distributed rate heterogeneity across sites and 1,000 bootstrap replicates.

### Time scaled Bayesian phylogenetic analysis

Bayesian phylogenetic analysis was performed using BEAST v2.5.2^41^ using the *Staphylococcus* core genome nucleotide alignments as input data. A HKY model of nucleotide substitution with four gamma-distributed rate heterogeneity across sites. A calibrated Yule process of speciation and a strict clock model as priors with all other default values was used. A single calibration point was used to infer a temporal scale, the divergence time between *Staphylococcus warneri* and *Staphylococcus pasteuri* estimated from the TimeTree database^30^ was used. The analysis was performed over three independent MCMC runs (10 million generations sampling every 1,000). Posterior distributions for parameter estimates and likelihood scores to approximate convergence were visualized with the Tracer program (v1.6.0^42^). Visual inspection of traces within and across runs, as well as the effective sample sizes ESS) of each parameter (> 200), allowed us to confirm that the analysis was adequately sampled. A maximum clade credibility (MCC) tree was chosen by TreeAnnotator (v1.8.1^41^) for each independent run after a 10% burn-in. MCC trees were visualized with FigTree v1.4.4.

## Supplementary information

Supplementary Information.

## Data Availability

Atomic coordinates have been deposited in the protein data bank (PDB) under the accession number 6QP1—*Staphylococcus hominis* PatB in the external aldimine in complex with l-cycloserine. 6QP2—*Staphylococcus hominis* PatB and 6QP3—crystal structure of the PLP-bound C-S lyase from *Bacillus subtilis* 168.

## References

[1] Shelley WB, Hurley HJ, Nichols AC (1953). Axillary odor; experimental study of the role of bacteria, apocrine sweat, and deodorants. AMA. Arch. Derm. Syphilol..

[2] Emter R, Natsch A (2008). The sequential action of a dipeptidase and a-lyase is required for the release of the human body odorant 3-methyl-3-sulfanylhexan-1-ol from a secreted Cys-Gly-(S) conjugate by corynebacteria. J. Biol. Chem..

[3] Bawdon D, Cox DS, Ashford D, James AG, Thomas GH (2015). Identification of axillary *Staphylococcus* sp. involved in the production of the malodorous thioalcohol 3-methyl-3-sufanylhexan-1-ol. FEMS Microbiol. Lett..

[4] Hodge BD, Brodell RT (2019). Anatomy, Skin Sweat Glands. StatPearls.

[5] Troccaz M (2015). Mapping axillary microbiota responsible for body odours using a culture-independent approach. Microbiome.

[6] Grice EA (2009). Topographical and temporal diversity of the human skin microbiome. Science.

[7] Egert M (2011). rRNA-based profiling of bacteria in the axilla of healthy males suggests right-left asymmetry in bacterial activity. FEMS Microbiol. Ecol..

[8] James AG, Hyliands D, Johnston H (2004). Generation of volatile fatty acids by axillary bacteria1. Int. J. Cosmet. Sci..

[9] James AG (2013). Microbiological and biochemical origins of human axillary odour. FEMS Microbiol. Ecol..

[10] James AG, Casey J, Hyliands D, Mycock G (2004). Fatty acid metabolism by cutaneous bacteria and its role in axillary malodour. World J. Microbiol. Biotechnol..

[11] Troccaz M, Starkenmann C, Niclass Y, van de Waal M, Clark AJ (2004). 3-Methyl-3-sulfanylhexan-1-ol as a major descriptor for the human axilla-sweat odour profile. Chem. Biodivers..

[12] Baumann T (2014). Glutathione-conjugated sulfanylalkanols are substrates for ABCC11 and γ-glutamyl transferase 1: a potential new pathway for the formation of odorant precursors in the apocrine sweat gland. Exp. Dermatol..

[13] Minhas GS (2018). Structural basis of malodour precursor transport in the human axilla. Elife.

[14] Callewaert C (2013). Characterization of staphylococcus and corynebacterium clusters in the human axillary region. PLoS ONE.

[15] Hiron A, Borezée-Durant E, Piard J-C, Juillard V (2007). Only one of four oligopeptide transport systems mediates nitrogen nutrition in *Staphylococcus aureus*. J. Bacteriol..

[16] Ferla MP, Patrick WM (2014). Bacterial methionine biosynthesis. Microbiology.

[17] Troccaz M, Benattia F, Borchard G, Clark AJ (2008). Properties of recombinant Staphylococcus haemolyticus cystathionine beta-lyase (metC) and its potential role in the generation of volatile thiols in axillary malodor. Chem. Biodivers..

[18] Auger S, Gomez MP, Danchin A, Martin-Verstraete I (2005). The PatB protein of *Bacillus subtilis* is a C-S-lyase. Biochimie.

[19] Berger BJ, English S, Chan G, Knodel MH (2003). Methionine regeneration and aminotransferases in Bacillus subtilis, Bacillus cereus, and Bacillus anthracis. J. Bacteriol..

[20] Zdych E, Peist R, Reidl J, Boos W (1995). MalY of *Escherichia coli* is an enzyme with the activity of a beta C-S lyase (cystathionase). J. Bacteriol..

[21] Miyazaki M (2006). A major urinary protein of the domestic cat regulates the production of felinine, a putative pheromone precursor. Chem. Biol..

[22] Percudani R, Peracchi A (2009). The B6 database: a tool for the description and classification of vitamin B6-dependent enzymatic activities and of the corresponding protein families. BMC Bioinform..

[23] Kezuka Y, Yoshida Y, Nonaka T (2012). Structural insights into catalysis by βC-S lyase from *Streptococcus anginosus*. Proteins Struct. Funct. Bioinform..

[24] Liang J, Han Q, Tan Y, Ding H, Li J (2019). Current advances on structure-function relationships of pyridoxal 5′-phosphate-dependent enzymes. Front. Mol. Biosci..

[25] Pearson AR, Mozzarelli A, Rossi GL (2004). Microspectrophotometry for structural enzymology. Curr. Opin. Struct. Biol..

[26] Ronda L (2011). Exploring methionine γ-lyase structure-function relationship via microspectrophotometry and X-ray crystallography. Biochim. Biophys. Acta Proteins Proteom..

[27] Lowther J (2010). Inhibition of the PLP-dependent enzyme serine palmitoyltransferase by cycloserine: evidence for a novel decarboxylative mechanism of inactivationwzy. Mol. BioSyst..

[28] Lowther J (2011). Role of a conserved arginine residue during catalysis in serine palmitoyltransferase. FEBS Lett..

[29] Singh R (2013). Chemogenomics of pyridoxal 5′-phosphate dependent enzymes. J. Enzyme Inhib. Med. Chem..

[30] Kumar S, Stecher G, Suleski M, Hedges SB (2017). TimeTree: a resource for timelines, timetrees, and divergence times. Mol. Biol. Evol..

[31] Pozzi L (2014). Primate phylogenetic relationships and divergence dates inferred from complete mitochondrial genomes. Mol. Phylogenet. Evol..

[32] Grice EA, Segre JA (2011). The skin microbiome. Nat. Rev. Microbiol..

[33] Council SE (2016). Diversity and evolution of the primate skin microbiome. Proc. R. Soc. B Biol. Sci..

[34] Starkenmann C, Niclass Y, Troccaz M, Clark AJ (2005). Identification of the precursor of (S)-3-methyl-3-sulfanylhexan-1-ol, the sulfury malodour of human axilla sweat. Chem. Biodivers..

[35] Brune I (2011). Identification of McbR as transcription regulator of aecD and genes involved in methionine and cysteine biosynthesis in Corynebacterium jeikeium K411. J. Biotechnol..

[36] Vagin A, Teplyakov A, IUCr (2010). Molecular replacement with *MOLREP*. Acta Crystallogr. Sect. D Biol. Crystallogr..

[37] Lebedev AA, Vagin AA, Murshudov GN (2008). Model preparation in *MOLREP* and examples of model improvement using X-ray data. Acta Crystallogr. Sect. D Biol. Crystallogr..

[38] Murshudov GN (2011). *REFMAC* 5 for the refinement of macromolecular crystal structures. Acta Crystallogr. Sect. D Biol. Crystallogr..

[39] Long F (2017). *AceDRG* : a stereochemical description generator for ligands. Acta Crystallogr. Sect. D Struct. Biol..

[40] Emsley P, Lohkamp B, Scott WG, Cowtan K (2010). Features and development of Coot. Biol. Crystallogr..

[41] Drummond, A. J. & Bouckaert, R. R. *Bayesian Evolutionary Analysis with BEAST* (Cambridge University Press, 2015). ISBN 978-1-107-01965-2.

[42] Rambaut A, Drummond AJ, Xie D, Baele G, Suchard MA (2018). Posterior summarization in Bayesian phylogenetics using Tracer 1.7. Syst. Biol..

