# Supplementary material for: The molecular basis of thioalcohol production in human body odour
Source: Sci Rep. 2020 Jul 27;10:12500. doi: 10.1038/s41598-020-68860-z (PMC7385124; doi:10.1038/s41598-020-68860-z)
Supplement: Supplementary file 1 — Supplementary Information. [file 41598_2020_68860_MOESM1_ESM.pdf]

The molecular basis of thioalcohol production in human body odour

Michelle Rudden[1,+], Reyme Herman[1,+], Matthew Rose[1], Daniel Bawdon[1], Diana S. Cox[2], Eleanor Dodson[3], Matthew T.G. Holden[4], Anthony J. Wilkinson[3], A. Gordon James[2], and Gavin H. Thomas[1]

[1]Department of Biology, University of York, Wentworth Way, York, YO10 5DD, UK

[2]Unilever R&D, Colworth Science Park, Sharnbrook, Bedford, MK44 1LQ, UK

[3]Department of Chemistry, University of York, Wentworth Way, York, YO10 5DD, UK

[4]School of Medicine, University of St Andrews, St Andrews, KY11 9TF, UK

[+]these authors contributed equally to this work

[*]For correspondence: gavin.thomas@york.ac.uk

SUPPLEMENTARY INFORMATION

FIGURES S1, S2, S3, S4, S5, S6, S7, S8, S9, S2, S10, S11, S12

TABLES S1,S2, S3, S4, S5, S6, S7, S8

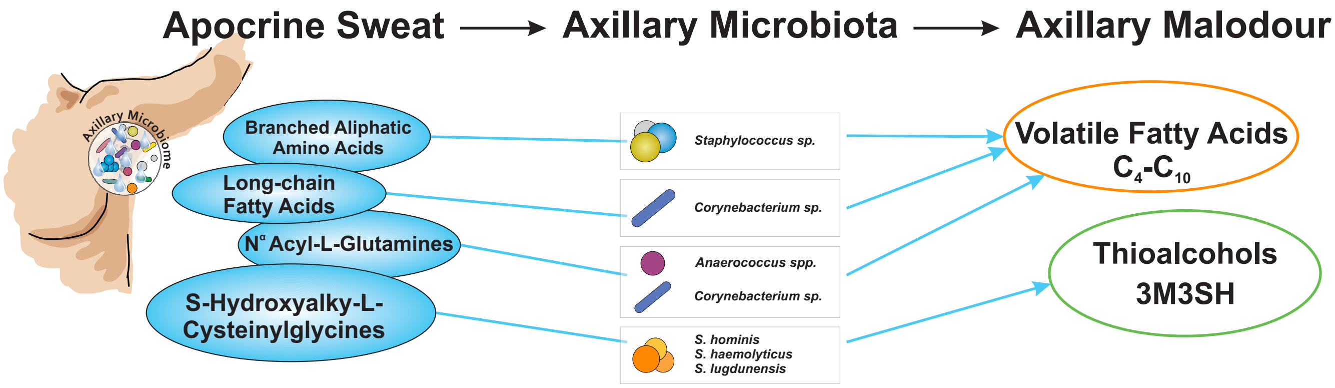

Figure S1 Biochemical origins of axillary malodour
Axillary malodour is composed of a mixture of volatile organic compounds. Volatile fatty acids (VFAs) and thioalcohols are considered the main causal agents of malodour[1]. The volatile compounds in axillary malodour are derived from the bacterial metabolism of odourless precursors secreted in apocrine sweat. *Corynebacterium sp.* produce structurally unusual medium chain acids from *N*-acylglutamine precursors. *Staphylococcus sp.* contribute by converting branched chain amino acids (such as leucine) into odorous short chain fatty acids (C_4-C_5). Malodour producing staphylococci such as *S. hominis* produce volatile sulphur thiols from dipeptide conjugated thiol precursors (S-hydroxyalkyl-L-cysteinlyglycines) that are secreted from the apocrine gland. These volatile thiols give BO its characteristic odour and are particularly pungent exhibiting a significantly lower olfactory threshold compared to other odorous volatiles in axillary malodour.

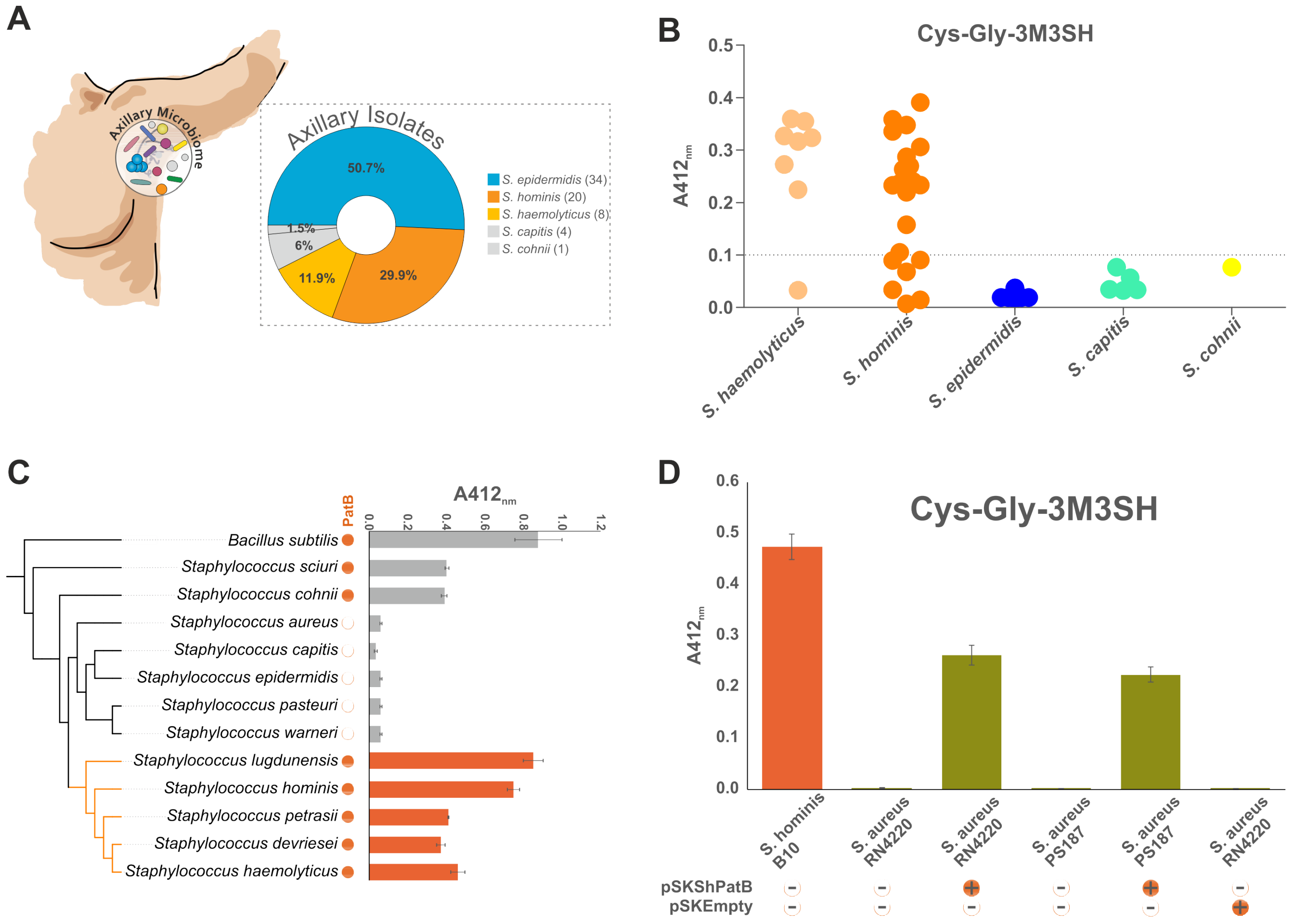

Figure S2 Bacterial biotransformation of Cys-Gly-3M3SH.

A. Overview of Staphylococcal sp. isolated from a Unilever axillary project. S. epidermidis is the most abundant staphylococcal species found in the axillary microbiota followed by S. hominis and S. haemolyticus. **B.** Axillary Staphylococcus isolates were screened for in vivo biotransformation of Cys-Gly-3M3SH. Whole cells were incubated at 37C overnight with Cys-3M3SH (2.5mM). Release of 3M3SH in the supernatant was detected by labelling with DTNB and measuring absorbance at 412nm (y-axis). Despite being the most dominant staphylococcal species in the underarm S. epidermidis lacks an equivalent ShPatB ortholog and does not metabolise Cys-Gly-3M3SH. In contrast, S. hominis and S. haemolyticus contain the PatB enzyme, yet several axillary isolates strains display differential in vivo activity toward Cys-Gly-3M3SH. **C.** In vivo biotransformation of Cys-Gly-3M3SH. Bacteria containing PatB enzymes are capable of metabolising the malodour precursor. Release of 3M3SH was labelled with DTNB and absorbance measured at 412nm. **D.** Non-malodour producing S. aureus can efficiently uptake and metabolise Cys-Gly-3M3SH upon overexpression of ShPatB. Wild type S. aureus does not contain a ShPatB ortholog and cannot metabolise Cys-3M3SH.Phylogenetic tree and bar chart was generated using iTOL (https://itol.embl.de/)

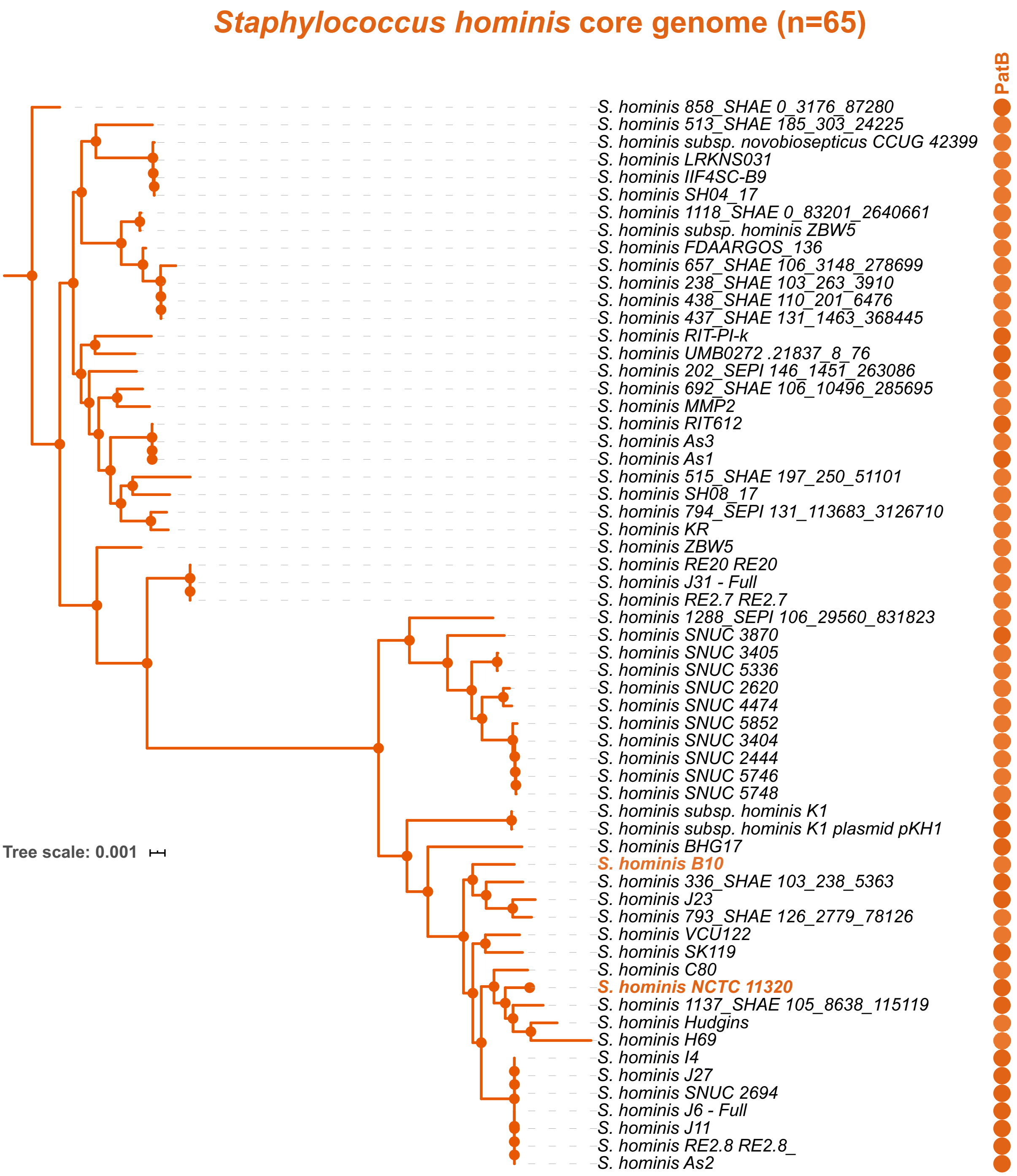

Figure S3 *S. hominis* Core Genome Phylogeny

ShPatB is a core gene present in *S. hominis* sp. Phylogenetic tree was generated using iTOL (https://itol.embl.de/)

	Cys-3M3SH	L-Felinine	Benzyl-Cysteine	L-Cystathione
BsPatB	306.89	141.6	305.65	50.35
CjPatB	137.88	189.6	398.40	331.37
SaPatB	78.53	74.7	142.22	112.65
SlPatB	304.25	0.0	387.76	7.35
SdPatB	670.27	*	231.56	2.65
ShPatB	765.98	1398.3	260.09	5.53
ShaPatB	734.81	*	409.38	5.88
EcMalY	15.09	*	146.46	208.09
SeMetC	0.08	3.1	0	42.91
ShMetC	1.40	0	1.45	122.60
SsPatB	0.40	*	0.34	0
ScPatB	14.09	*	10.76	3.44

K_{cat}/K_M mM^{-1} min^{-1}

High Activity

Low Activity

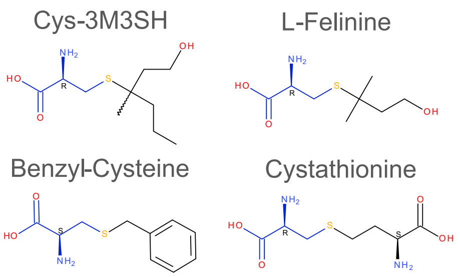

Figure S4 ShPatB prefers Cys-3M3SH and related substrates.
Comparison of catalytic efficiencies (Kcat/KM) for selected PLP dependent C-S -lyases against various cysteine-S-conjugate substrates. Malodour producing staphylococci have the highest activity toward Cys-3M3SH compared to other substrates

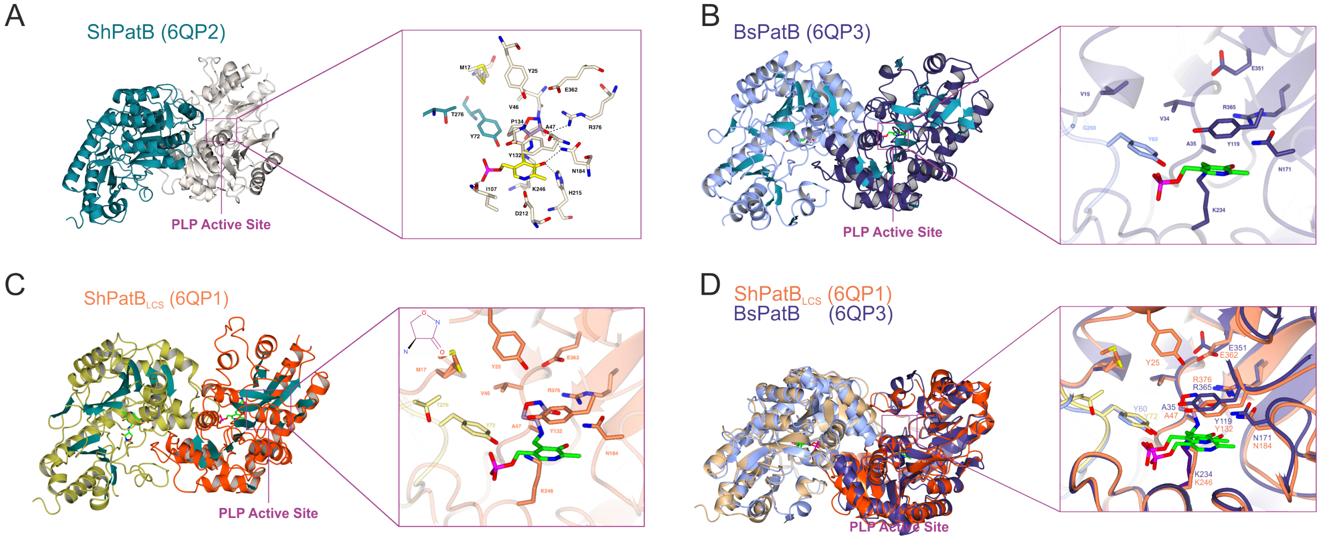

Figure S5 Overall architecture of ShPatB and BsPatB PLP dependent C-S--lyases.
A. Crystal structure of ShPatB (PDB 6QP2) with zoomed view of PLP bound in the internal aldimine form to K246. **B.** Crystal structure of PatB from Bacillus subtilis (PDB 6QP3) showing internal aldimine of PLP covalently bound to K234 in active site of BsPatB. **C.** Structure of ShPatB bound in complex with L-cycloserine (PDB ID 6QP1). L-cycloserine is shown in the external aldimine form bound to PLP. **D.** Superposition of the ShPatBLCS (orange) and BSPatB (blue) done using the SSM superpose algorithm on CCP4MG with a calculated RMSD of 1.31. All structural images were generated in CCP4MG (http://www.ccp4.ac.uk/MG/)

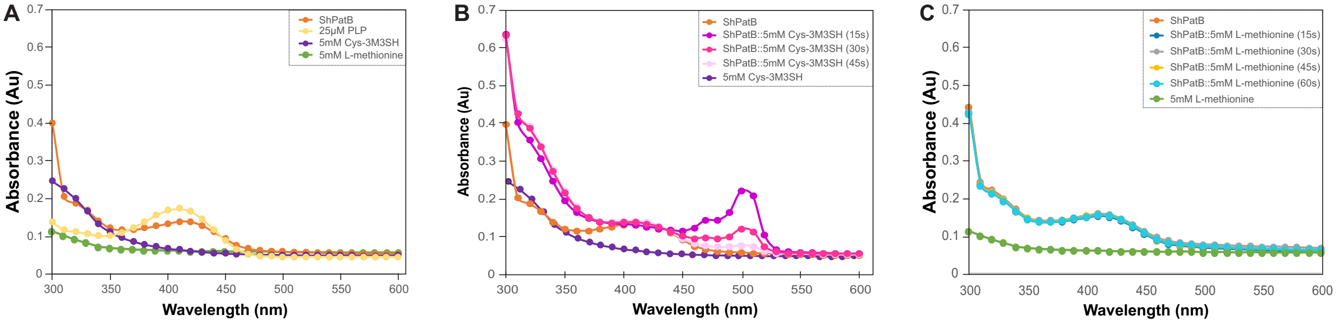

Figure S6 UV-Vis Absorption spectra of wild-type ShPatB in the presence of Cys-3M3SH
A. Absorption spectra of ShPatB, PLP and substrates only. Presence of the PLP cofactor observed at peak 410nm. **B.** Spectral changes of ShPatB in the presence of 5mM Cys-3M3SH. ShPatB with Cys-3M3SH shows a peak at 500nm concomitant with a slight decrease in absorption at 410nm (PLP). At later timepoints, the peak at 500nm subsequently decreases and the peak at 410nm returned to the level of wildtype ShPatB. **C.** Absorption spectra of ShPatB in the presence of L-methionine. No additional peaks were observed with L-methionine, a methionine gamma-lyase substrate.

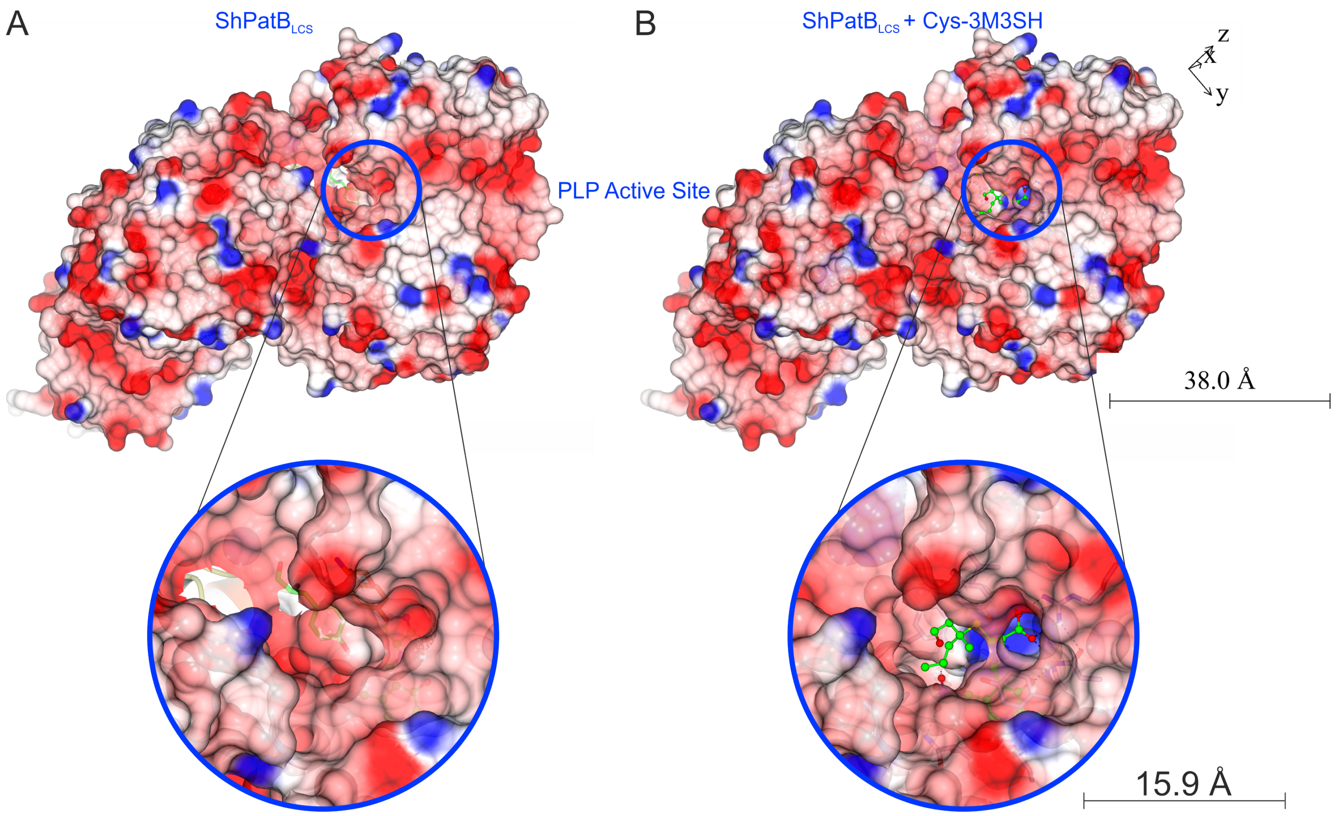

Figure S7 Modelled Cys-3M3SH extends toward the protein surface
A. Electrostatic surface potential of ShPatB$_{LCS}$ and **B.** electrostatic surface potential of ShPatB$_{LCS}$ with Cys-3M3SH modelled into the binding site. The side group of Cys-3M3SH extends outward toward the protein surface from the recessed binding site. Electrostatic surfaces were generated by CCP4MG. Colours represent charge density blue (positive) and red (negative). Images were generated in CCP4MG (http://www.ccp4.ac.uk/MG/)

/

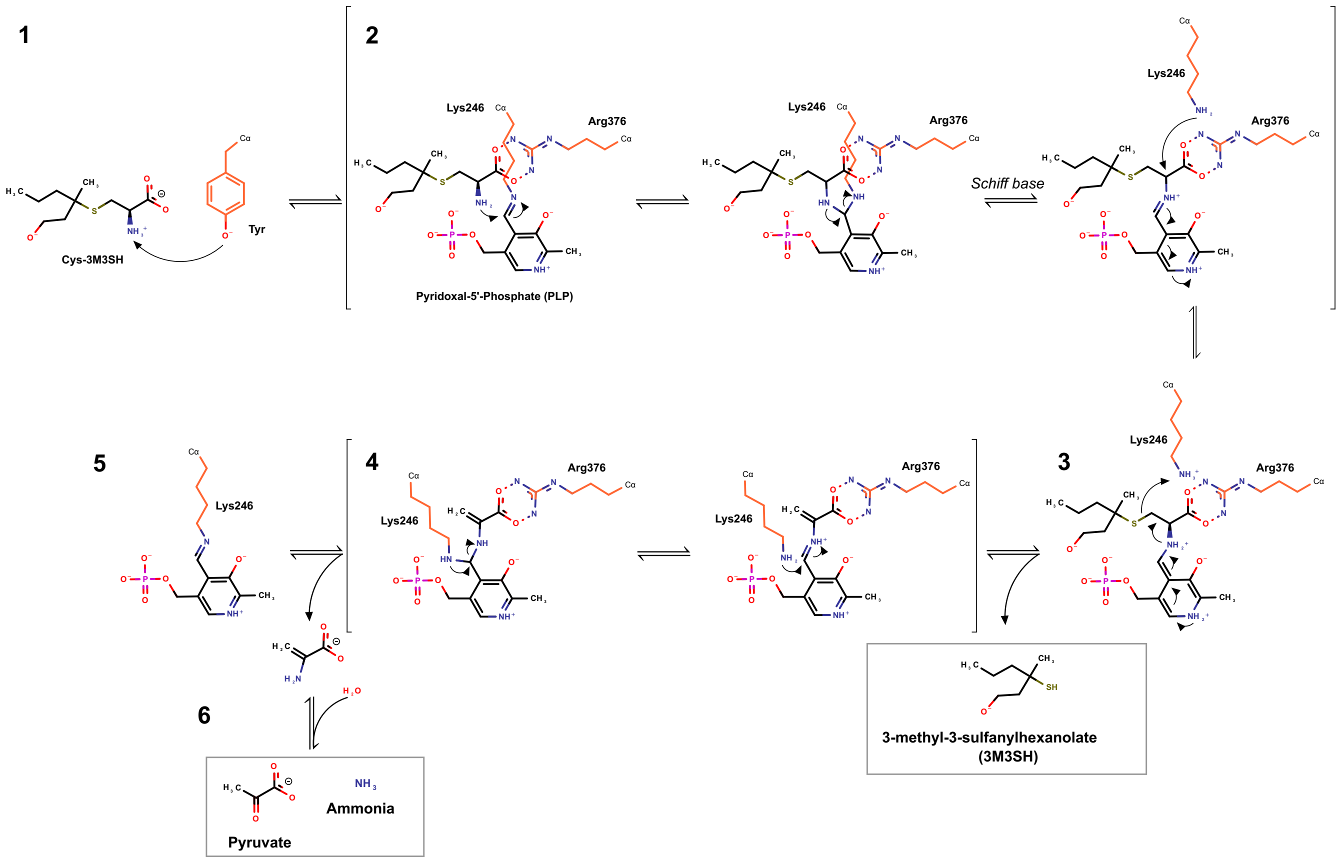

Figure S8 Suggested mechanism of Cys-3M3SH cleavage by ShPatB
Residues are shown in orange. **(1)** A conserved tyrosine residue within the binding pocket deprotonates the amino group on Cys-3M3SH. **(2)** The deprotonated amine group carries out a nucleophilic attack on the PLPLys246 bond to release Lys246 and form a Schiff base with the PLP. **(3)** The free Lys246 can now accept the proton via the sulphur causing the C–S bond breakage and the release of the odorous molecule, 3-methyl-3-sulfanylhexanolate (3M3SH). **(4)** The lysine is able to carry out a nucleophilic attack on the external aldimine, **(5)**releasing the internal aldimine and (6) -aminoacrylate which is tautomerized and hydrolysed to form pyruvate and ammonia.

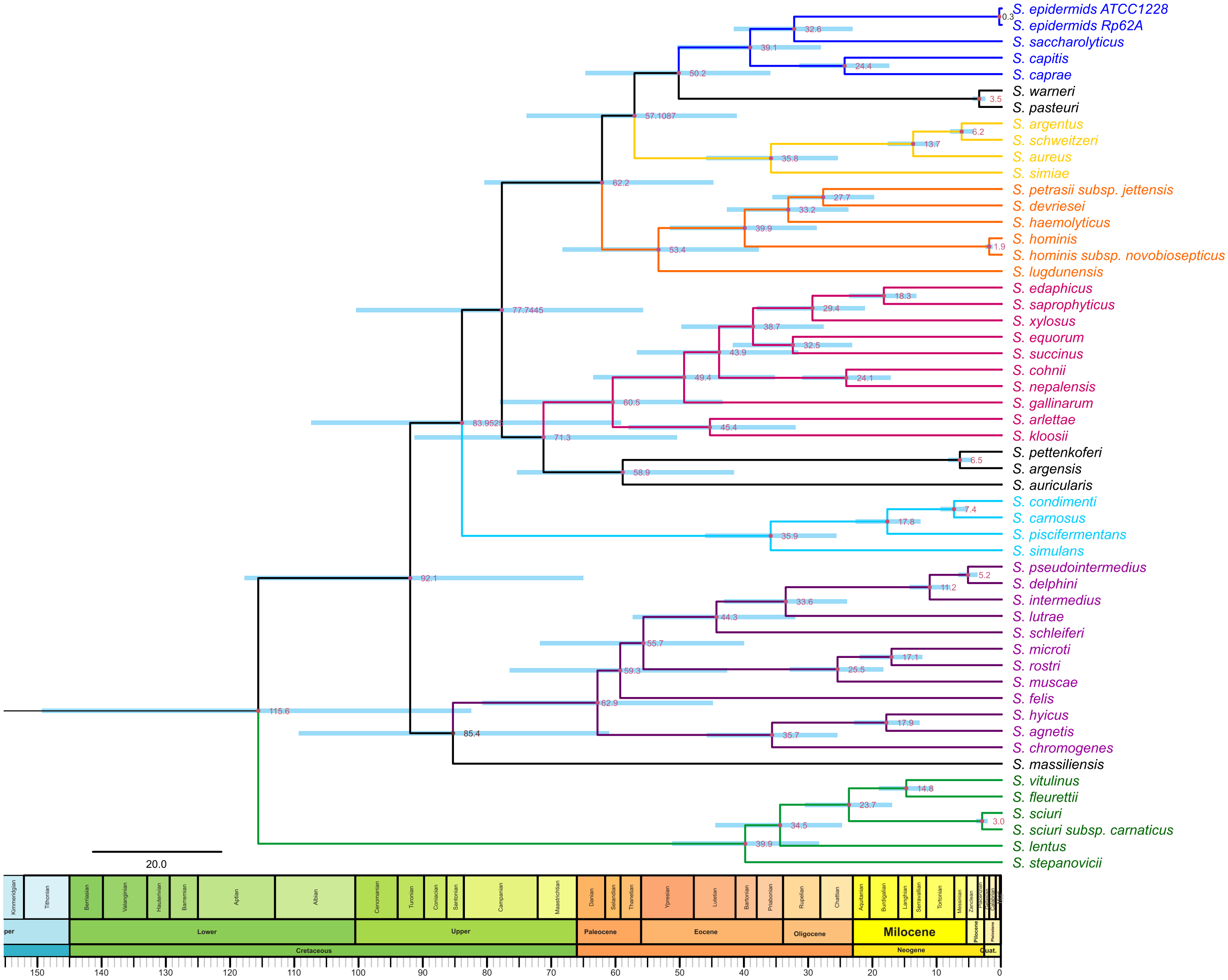

Figure S9 Estimated divergence time for *Staphylococcus* spp.
Expanded phylogenetic tree showing all estimated divergence times for *Staphylococcus* spp. Divergence confidence intervals are drawn as blue scale bars around the respective nodes (represented by pink dots). Phylogenetic tree was generated using FigTree V1.4.4.

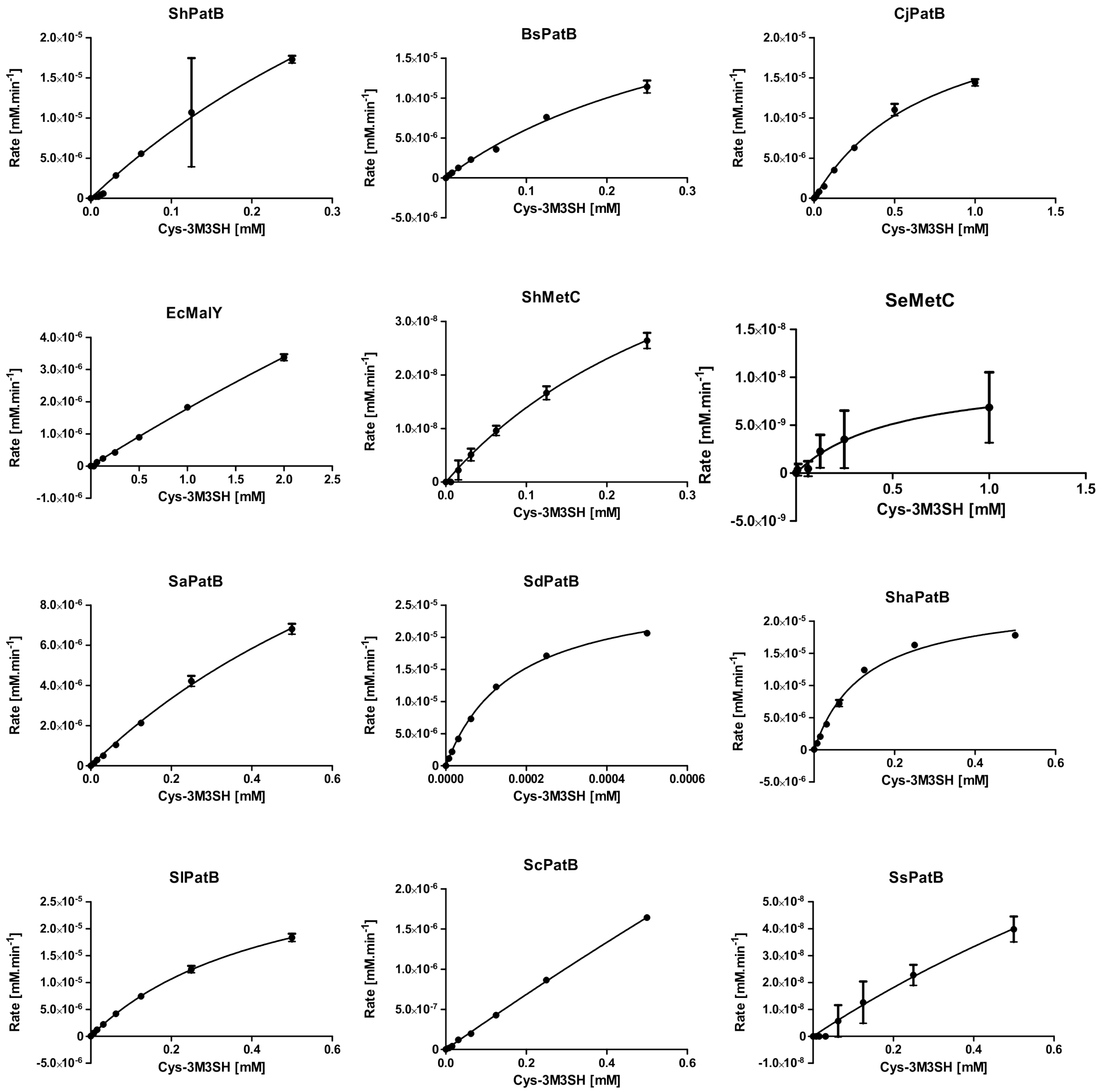

Figure S10 Michaelis-Menten plots for biotransformation of Cys-3M3SH by various C-S lyases.
Dots represent experimental points, lines are Michaelis-Menten fit

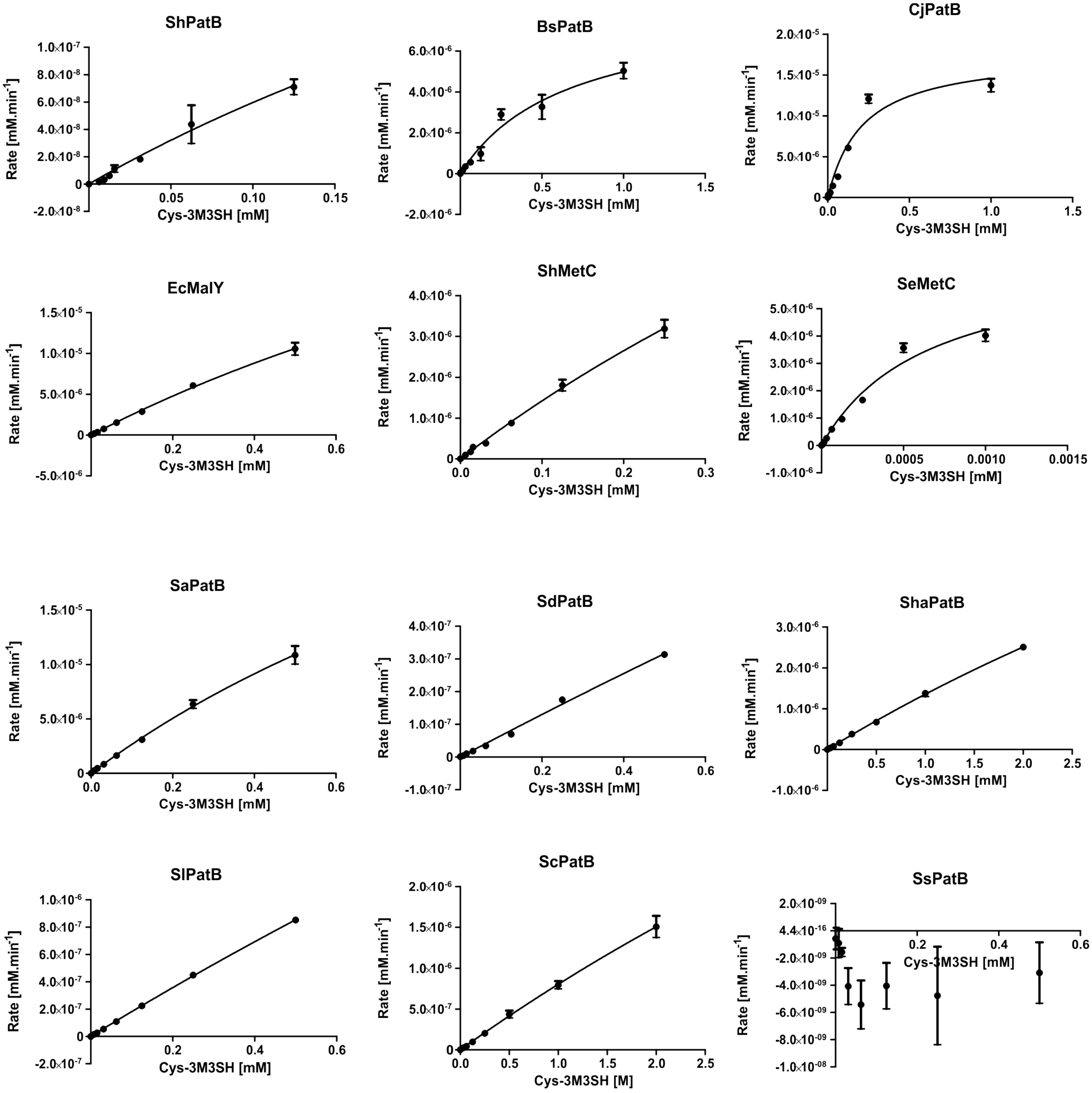

Figure S11 Michaelis-Menten plots for biotransformation of Cystathionine by various C-S lyases.
Dots represent experimental points, lines are Michaelis-Menten fit

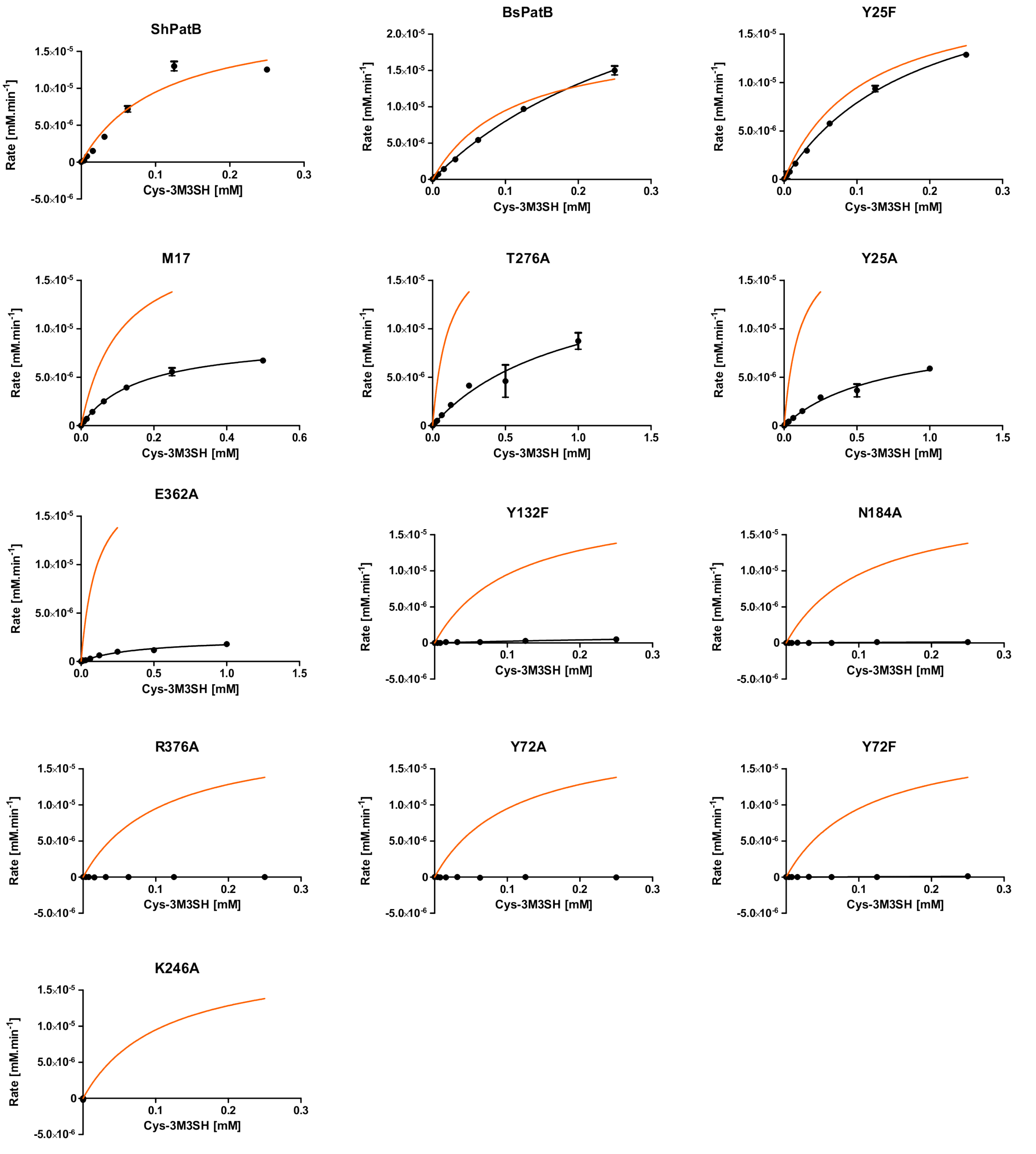

Figure S12 Michaelis-Menten plots for biotransformation of Cys-3M3SH by various ShPatB mutants. ShPatB wild-type (orange line) is shown in each plot for comparison. Dots represent experimental points, lines are Michaelis-Menten fit

Supplementary Data Tables

Kinetic characterisation of C-S-β-lyases with various cysteine-S-conjugate ligands.

Table S1| Cys-3M3SH

Enzyme	μM	K_M(mM)	K_M[95% CI]	k_{cat}(min$^{-1)}$	K_{cat}[95% CI]	k_{cat}/K_M(mM^{-1}min^{-1})	R^2
ShPatB	0.25	0.11	[0.02, 0.20]	74.47	[47.2, 101.7]	670.90	0.970
ShaPatB	0.25	0.13	[0.08, 0.17]	93.10	[80.15, 106.10]	734.81	0.991
SdPatB	0.25	0.17	[0.14, 0.19]	111.60	[104.80, 118.40]	670.27	0.999
BsPatB	0.25	0.38	[0.07, 0.41]	115.30	[76.73, 153.80]	306.89	0.996
SlPatB	0.25	0.47	[0.45, 0.49]	141.90	[138.30, 145.50]	304.25	1.000
CjPatB	0.25	0.74	[0.52, 0.97]	102.60	[85.66, 119.50]	137.88	0.996
SaPatB	0.25	1.16	[0.69, 1.63]	91.10	[63.11, 119.10]	78.53	0.998
EcMalY	0.24	17.49	[2.53, 32.45]	264.00	[28.89, 235.10]	15.09	0.999
ScPatB	0.25	7.09	[-0.10, 14.27]	99.85	[4.27, 195.40]	14.09	1.000
EcTnaA	1.00	2.15	[1.08, 3.22]	3.06	[2.12, 4.00]	1.42	0.993
ShMetC	0.125	0.38	[0.22, 0.54]	0.53	[0.38, 0.69]	1.40	0.998
SsPatB	0.25	2.09	[-1.19, 5.36]	0.83	[-0.26, 1.92]	0.40	0.991
SeMetC	0.25	0.53	[0.13, 0.92]	0.04	[0.03, 0.06]	0.08	0.984

Table S2| L-Cystathionine

Enzyme	μM	K_M(mM)	K_M[95% CI]	k_{cat}(min^{-1})	K_{cat}[95% CI]	k_{cat}/K_M($mM^{-1}min^{-1}$)	R^2
CjPatB	0.25	0.21	[0.057,0.37]	70.88	[50.34,91.42]	331.37	0.954
EcMalY	0.125	2.2	[1.06,3.34]	457.8	[257.2,658.5]	208.09	0.999
ShMetC	0.125	1.27	[0.31,2.22]	155.7	[54.76,256.6]	122.6	0.998
SaPatB	0.25	1.72	[1.04,2.39]	193.3	[131.5,255.1]	112.65	0.999
BsPatB	0.25	0.66	[0.21,1.10]	33.01	[21.28, 44.73]	50.35	0.978
SeMetC	0.25	0.65	[0.22,1.08]	27.94	[18.21,37.67]	42.91	0.979
SlPatB	0.25	6.52	[3.10,9.94]	47.92	[24.28,71.55]	7.35	1
ShaPatB	0.25	11.7	[6.51,16.94]	68.93	[42.01,95.86]	5.88	1
ShPatB	0.125	0.63	[-0.55,1.81]	3.47	[-2.14,9.08]	5.53	0.989
ScPatB	0.25	14.2	[7.33,21.07]	48.83	[27.67,70.00]	3.44	1
SdPatB	0.25	10.39	[-37.02,57.80]	27.55	[-93.22,148.3]	2.65	0.996
EcTnaA	1	*	-	*	-	*	-
SsPatB	0.25	*	-	*	-	*	-

Table S3| | L-Felinine

Enzyme	μM	K_M(mM)	K_M[95% CI]	k_{cat}(min^{-1})	K_{cat}[95% CI]	k_{cat}/K_M(mM^{-1}min^{-1})	R^2
ShPatB	0.125	0.1	[0.011,0.28]	200.1	[105.7,294.3]	1398.3	0.9561
CjPatB	0.25	0.8	[0.74,0.93]	158.8	[148.7,168.9]	189.6	1.000
BsPatB	0.25	0.4	[0.20,0.60]	56.7	[37.51,75.85]	141.6	0.996
SaPatB	0.25	1.1	[0.67,1.50]	81.2	[58.19,104.2]	74.7	0.998
EcMalY	0.125	*	-	*	-	*	-
SeMetC	0.25	4.7	[2.48,7.00]	14.6	[8.634, 20.49]	3.1	0.999
ShMetC	0.125	*	-	*	-	*	-
EcTnaA	1	3.3	[0.55,5.97]	0.7	[0.3042,1.131]	0.2	0.989
SdPatB	*	-	-	-	-	-	-
ShaPatB	*	-	-	-	-	-	-
SlPatB	*	-	-	-	-	-	-
ScPatB	*	-	-	-	-	-	-
SsPatB	*	-	-	-	-	-	-

Table S4| Steady state kinetics for ShPatB variants for Cys-3M3SH catalysis.

Enzyme	K_M(mM)	k_{cat}(min^{-1})	k_{cat}/K_M(mM^{-1}min^{-1})	R^2
ShPatB	0.11	77.09	699.55	0.952
Y25F	0.19	90.88	485.21	0.999
BsPatB	0.36	146.4	409.63	0.999
M17A	0.16	44.83	278.97	0.999
T276A	0.97	66.06	68.34	0.975
Y25A	0.69	38.88	56.06	*
E362A	0.42	9.804	23.42	0.984
Y132F	0.48	5.822	12.04	0.952
N184A	0.52	1.774	3.14	0.752
K246A	*	*	*	*
R376A	*	*	*	*
Y72A	*	*	*	*
Y72F	*	*	*	*

Table S5| Data collection, phasing and refinement statistics for all structures in this study.

	**ShPatB$_{LCS}$**	**ShPatB**	**BsPatB**
**PDB**	6QP1	6QP2	6QP3
**Source and Beamline**	Diamond i03	Diamond i03	Diamond i04
**Wavelength**	0.9762	0.9763	0.9795
**Type**	Synchrotron	Synchrotron	Synchrotron
**Detector**	Dectris Pilatus	Dectris Pilatus	Dectris Pilatus
**Data Statistics**			
**Space group**	$P2_12_12_1$	$P2_12_12_1$	$P2_12_12_1$
**Cell constants a, b, c (Å)**	55.47, 115.46, 118.96	54.75, 113.63, 118.10	75.28, 105.53, 209.58
**Cell constants α, β, γ (°)**	90.00,90.00,90.00	90.00,90.00,90.00	90.00, 90.00,90.00
**Overall resolution range (Å)**	51.94 - 1.42	52.40 - 1.60	61.28 - 2.30
**Inner shell resolution range (Å)**	51.94 - 7.78	52.40 - 8.76	61.28 - 11.26
**Outer shell resolution range (Å)**	1.44 - 1.42	1.63 - 1.60	2.35 - 2.30
**Overall completeness (%)**	99.5	100	96
**R_{merge} for all I+ and I-[a]**	0.103	0.098	0.197
**< I/σ(I) >**	1.34	1.35	1.65
**Overall multiplicity**	7.9	8	7.9
**Refinement Statistics**			
**Program**	REFMAC	REFMAC	REFMAC
**Number of unique reflections**	143787	97859	72018
**Number of reflections, Free R set**	7085 (4.93% overall)	4990 (5.10% overall)	3601 (5% overall)
**R, Rfree [b,c]**	0.200, 0.226	0.201, 0.243	0.215, 0.260
**Total number of atoms**	6889	6686	12174
**Average B, all atoms (A$\hat{2}$)**	23	30	29
**RMS Deviations – Bonds[d]**	0.0118	0.0195	0.0087
**RMS Deviations – Angles[d]**	1.625	1.364	1.652
**Ramachandran plot[e]**	0.1%	0.0%	0.3%

[a]Rmerge = $\sum_{HKL}\sum_{I}|I_I$ - <I> $|/\sum_{HKL}\sum_{I}$ <I> where I_I is the intensity of the ith measurement of a reflection with indexes hkl and I is the STATISTICALLY WEIGHTED AVERAGE REFLECTION INTENSITY.

[b] R-factor =$||Fo| - |Fo||/|Fo|$ where Fo and Fc are the observed and calculated structure factor amplitudes, respectively.

[c]R-free is the R-factor calculated with 5 % of the reflections chosen at random and omitted from refinement.

[d]Root-mean-square deviation of bond lengths and bond angles from ideal geometry.

[e]Percentage of residues in outlier regions.

Table S6| List of bacterial strains used in this study.

Strain	Genotype	Reference
Escherichia coli MC1061	F– λ– δ(ara-leu)7697 [araD139]B/r δ(codB-lacI)3 galK16 galE15 e14– mcrA0 relA1 rpsL150(StrR) spoT1 mcrB1 hsdR2(r–m+)	2
Escherichia coli BW25113	(araD-araB)567 (rhaD-rhaB)568 lacZ4787 (::rrnB-3) hsdR514 rph-1	3
S. aureus NCTC 8325	Wild-type reference strain	4
S. aureus RN4220	Restriction deficient derivative of 8325, capable of accepting plasmids from *E. coli*	5
S. epidermidis Rp62a	Wild-type reference strain	6
Bacillus subtilis 168	Reference strain, *trpC2*	7
S. hominis B10	Skin isolated strain	University of Liverpool
Corynebacterium jekieum K411	Wild-type reference strain	8
Staphylococcus lugdunensis HKU0901	Wild-type reference strain	9
S. haemolyticus JCSC1435	Wild-type reference strain	10
S. capitis	20326, DSM Type strain	11
S. hominis subsp. novobiosepticus	15614, DSM Type strain	12
S. sciuri	20345, DSM Type strain	13
S. pasteuri	10656, DSM Type strain	14
S. warneri	20260, DSM Type strain	15
S. cohnii	20260, DSM Type strain	15
S. devriesei	25293, DSM Type strain	16

/

Table S7| List of plasmids used in this study.

Plasmid	Description	Accession No.	Reference
pBAD	L-arabinose inducible expression vector, Amp^R, His$_{10}$	-	17
pBAD::EcMalY	MalY protein from *E. coli* BW25113	P23256	This study
pBAD::ShMetC	MetC protein from S. hominis B10	WP_077700742	This study
pBAD::SeMetC	MetC protein from *S. epidermidis* Rp62a	Q5HRU7	This study
pBAD::ShPatB	PatB protein from *S. hominis* B10	WP_077700575.1	This study
pBAD::BsPatB	PatB protein from *B. subtilis* 168	Q08432	This study
pBAD::CjAecD	PatB protein from *C. jekieum* K411	Q4JWQ6	This study
pBAD::SlPatB	PatB protein from *S. lugdunensis* HKU09-01	ADC86375.1	This study
pBAD::SdPatB	PatB protein from *S. devreisei*	WP_103167498	This study
pBAD::ShaPatB	PatB protein from*S. haemolyticus* JCSC1435	Q4L9Z5	This study
pBAD::SsPatB	PatB protein from *S. sciuri*	WP$_0$75577893	This study
pBAD::ScPatB	PatB protein from *S. cohnii*	WP_107523385	This study
pBAD::SaPatB	PatB protein from *S. anginosus* IMU102	A6BMJ3	This study
pSK5632	*E.coli* - *S. aureus* expression shuttle vector	-	18
pSKShPatB	pSK5632 containing PatB from *S. hominis* B10	WP_077700575.1	This study

Table S8| List of staphylococci genomes used for phylogenetic analyses.

Organism	Genbank Accession No.
S. agnetis	NZ_CP009623
S. argensis	NZ_PPPX01000001.1
S. argentus	NC_016941.1
S. arlettae	NZ_ALWK01000001.1
S. aureus NCTC 8325	NC_A1:B53007795.1
S. auricularis	NZ_LLER01000001
S. capitis	NZ_CP007601
S. caprae	NZ_AP018585
S. carnosus	NC_012121.1
S. chromogenes	NZ_PPQK01000001
S. cohnii	NZ_LATV01000015
S. condimenti	NZ_PPQY01000001.1
S. delphini	NZ_LR134263
S. devriesei	NZ_PYZJ01000001.1
S. edaphicus	NZ_MRZN01000010.1
S. epidermids ATCC 1228	NC_004461.1
S. epidermids Rp62A	NC_002976.31
S. equorum	NZ_CP013114.1
S. felis	NZ_CP027770
S. fleurettii	NZ_PYYE01000001.1
S. gallinarum	NZ_JXCF01000004.1
S. hyicus	NZ_CP008747
S. haemolyticus	NC_007168.1
S. hominis	NZ_GL545252
S. hominis subsp.novobiosepticus	NZ_PPQX01000001.1
S. kloosii	NZ_CP027846
S. lentus	NZ_AJXO01000001
S. lugdunensis	NC_013893.1_1
S. lutrae	NZ_CP020773
S. massiliensis	NZ_AMSQ01000001.1
S. microti	NZ_JXWY01000001
S. muscae	NZ_CP027848
S. nepalensis	NZ_CP017460.1
S. pasteuri	NC_022737.1
S. petrasii subsp.jettensis	NZ_PPQT01000001.1
S. pettenkoferi	NZ_AGUA01000116
S. piscifermentans	NZ_LT906447.1
S. pseudointermedius	NC_014925
S. rostri	NZ_PPRF01000001
S. saccharolyticus	NZ_UHDZ01000002.1
S. saprophyticus	NC_007350.1
S. schleiferi	NZ_CP009470.1
S. schweitzeri	NZ_LR134304.1
S. sciuri	NZ_CP022046.2
S. simiae	NZ_AEUN01000565
S. simulans	NZ_CP014016.2
S. stepanovicii	NZ_LT906462.1
S. succinus	NZ_CP018199.1
S. vitulinus	NZ_AJTR01000001.1
S. warneri	NC_020164.1
S. xylosus	NZ_CP007208.1

References

1. James, A. G. *et al.* Microbiological and biochemical origins of human axillary odour. *FEMS microbiology ecology* **83**, 527–40, DOI: 10.1111/1574-6941.12054 (2013).
2. Casadaban, M. J. & Cohen, S. N. Analysis of gene control signals by DNA fusion and cloning in Escherichia coli. *J. Mol. Biol.* **138**, 179–207, DOI: 10.1016/0022-2836(80)90283-1 (1980).
3. Baba, T. *et al.* Construction of Escherichia coli K-12 in-frame, single-gene knockout mutants: the Keio collection. *Mol. systems biology* **2**, 2006.0008, DOI: 10.1038/msb4100050 (2006).
4. Gillaspy A.F. *et al. The Staphylococcus aureus NCTC8325 Genome*, vol. null (2006).
5. Nair, D. *et al.* Whole-Genome Sequencing of Staphylococcus aureus Strain RN4220, a Key Laboratory Strain Used in Virulence Research, Identifies Mutations That Affect Not Only Virulence Factors but Also the Fitness of the Strain. *J. Bacteriol.* **193**, 2332–2335, DOI: 10.1128/JB.00027-11 (2011).
6. Gill, S. R. *et al.* Insights on evolution of virulence and resistance from the complete genome analysis of an early methicillin-resistant Staphylococcus aureus strain and a biofilm-producing methicillin-resistant Staphylococcus epidermidis strain. *J. Bacteriol.* **187**, 2426–2438, DOI: 10.1128/JB.187.7.2426-2438.2005 (2005).
7. Kunst, F. *et al.* The complete genome sequence of the gram-positive bacterium Bacillus subtilis. *Nature* **390**, 249–256, DOI: 10.1038/36786 (1997).
8. Tauch, A. *et al.* Complete Genome Sequence and Analysis of the Multiresistant Nosocomial Pathogen Corynebacterium jeikeium K411, a Lipid-Requiring Bacterium of the Human Skin Flora. *J. Bacteriol.* **187**, 4671–4682, DOI: 10.1128/JB.187.13.4671-4682.2005 (2005).
9. Tse, H. *et al.* Complete genome sequence of Staphylococcus lugdunensis strain HKU09-01. *J. Bacteriol.* **192**, 1471–1472, DOI: 10.1128/JB.01627-09 (2010).
10. Takeuchi, F. *et al.* Whole-genome sequencing of Staphylococcus haemolyticus uncovers the extreme plasticity of its genome and the evolution of human-colonizing staphylococcal species. *J. Bacteriol.* **187**, 7292–7308, DOI: 10.1128/JB.187.21.7292-7308.2005 (2005).
11. Kloos, W. E. & Schleifer, K. H. Isolation and characterization of Staphylococci from human skin. II. Descriptions of four new species: Staphylococcus warneri, Staphylococcus capitis, Staphylococcus hominis, and Staphylococcus simulans. *Int. J. Syst. Bacteriol.* **25**, 62–79, DOI: 10.1099/00207713-25-1-62 (1975).
12. Kloos, W. E. *et al.* Staphylococcus hominis subsp. novobiosepticus subsp. nov., a novel trehalose- and N-acetyl-D-glucosamine-negative, novobiocin- and multiple- antibiotic-resistant subspecies isolated from human blood cultures. *Int. J. Syst. Bacteriol.* **48**, 799–812, DOI: 10.1099/00207713-48-3-799 (1998).
13. Kloos, W. E., Schleifer, K. H. & Smith, R. F. Characterization of Staphylococcus sciuri sp. nov. and its subspecies. *Int. J. Syst. Bacteriol.* **26**, 22–37, DOI: 10.1099/00207713-26-1-22 (1976).
14. Chesneau, O., Morvan, A., Grimont, F., Labischinski, H. & El Solh, N. Staphylococcus pasteuri sp. nov., isolated from human, animal, and food specimens. *Int. J. Syst. Bacteriol.* **43**, 237–244, DOI: 10.1099/00207713-43-2-237 (1993).
15. Schleifer, K. H. & Kloos, W. E. Isolation and characterization of Staphylococci from human skin. I. Amended descriptions of Staphylococcus epidermidis and Staphylococcus saprophyticus and descriptions of three new species: Staphylococcus cohnii, Staphylococcus haemolyticus, and Staphylococcus xylosus. *Int. J. Syst. Bacteriol.* **25**, 50–61, DOI: 10.1099/00207713-25-1-50 (1975).
16. Supré, K. *et al.* Staphylococcus devriesei sp. nov., isolated from teat apices and milk of dairy cows. *Int. J. Syst. Evol. Microbiol.* **60**, 2739–2744, DOI: 10.1099/ijs.0.015982-0 (2010).
17. Guzman, L. M., Belin, D., Carson, M. J. & Beckwith, J. Tight regulation, modulation, and high-level expression by vectors containing the arabinose P(BAD) promoter. *J. Bacteriol.* **177**, 4121–4130, DOI: 10.1128/jb.177.14.4121-4130.1995 (1995).
18. Grkovic, S. *et al.* Stable low-copy-number Staphylococcus aureus shuttle vectors. DOI: 10.1099/mic.0.25951-0 (2003).
